# N^6^ ‐methyladenosine‐modified circRNA RERE modulates osteoarthritis by regulating β‐catenin ubiquitination and degradation

**DOI:** 10.1111/cpr.13297

**Published:** 2022-06-22

**Authors:** Yuxi Liu, Yunhan Yang, Yucheng Lin, Bing Wei, Xinyue Hu, Li Xu, Weituo Zhang, Jun Lu

**Affiliations:** ^1^ Department of Orthopaedic Surgery Zhongda Hospital, School of Medicine, Southeast University Nanjing Jiangsu China; ^2^ School of Life Science and Technology Southeast University Nanjing Jiangsu China

## Abstract

**Objectives:**

N^6^‐methyladenosine (m6A) is one of the most abundant internal RNA modifications. We investigated the role of m6A‐modified circRERE in osteoarthritis (OA) and its mechanism.

**Materials and Methods:**

CircRERE and *IRF2BPL* were screened by microarrays. The role of m6A‐modification in circRERE was examined by methylated RNA precipitation and morpholino oligo (MOs) treatment. The axis of circRERE/miR‐195‐5p/IRF2BPL/β‐catenin was determined using flow cytometry, western blotting and immunofluorescence in human chondrocytes (HCs) and corroborated using a mouse model of destabilization of medial meniscus (DMM) with intra‐articular (IA) injection of adeno‐associated viruses (AAV).

**Results:**

CircRERE was decreased in OA cartilage and chondrocytes compared with control. CircRERE downregulation was likely attributed to its increased m6A modification prone to endoribonucleolytic cleavage by YTHDF2‐HRSP12‐RNase P/MRP in OA chondrocytes. MOs transfection targeting HRSP12 binding motifs in circRERE partially reversed decreased circRERE expression and increased apoptosis in HCs treated with IL‐1β for 6 h. CircRERE exerted chondroprotective effects by targeting miR‐195‐5p/IRF2BPL, thus regulating the ubiquitination and degradation of β‐catenin. CircRere (mouse homologue) overexpression by IA‐injection of AAV‐circRere into mice attenuated the severity of DMM‐induced OA, whereas AAV‐miR‐195a‐5p or AAV‐sh‐Irf2bpl reduced the protective effects. The detrimental effects of AAV‐sh‐Irf2bpl on DMM‐induced OA were substantially counteracted by ICG‐001, an inhibitor of β‐catenin.

**Conclusions:**

Our study is a proof‐of‐concept demonstration for targeting m6A‐modified circRERE and its target miR‐195‐5p/IRF2BPL/β‐catenin as potential therapeutic strategies for OA treatment.

## Graphical abstract

Liu et al. demonstrate that circRERE expression is downregulated in human osteoarthritis (OA) cartilage and chondrocytes, and circRERE downregulation is likely attributed to its increased m6A modification prone to endoribonucleolytic cleavage by YTHDF2‐HRSP12‐RNase P/MRP. Mechanistically, circRERE downregulation leads to aberrant β‐catenin ubiquitin and degradation by targeting miR‐195‐5p/IRF2BPL during OA pathogenesis.

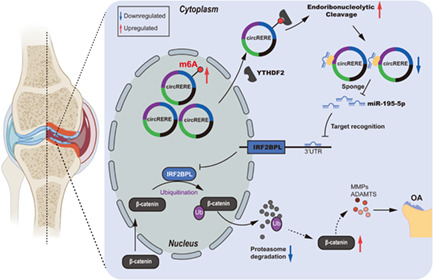

## 1 INTRODUCTION

Osteoarthritis (OA) is a common debilitating joint disease characterized by cartilage destruction, synovial inflammation, and bone remodelling in the form of subchondral bone thickening and osteophyte formation, eventually leading to chronic joint pain and disability.^1^, ^2^ The risk factors for OA include age, obesity, injury, and genetics.^3^ OA pathogenesis is complicated and multifactorial involving inflammation, cellular senescence, mitochondrial dysfunction, aberrant epigenetic regulation, and alterations in signalling pathways, such as Wnt/β‐catenin.^4^, ^5^, ^6^ OA represents a significant source of morbidity and socioeconomic burden as aging and obese populations are increasing worldwide.^7^, ^8^ The current pharmaceutical treatment for OA mainly refers to symptomatic relief rather than stopping or delaying its progression.^9^ Despite the progressive development of OA diagnosis using imaging equipment or biochemical markers, it is difficult to detect early‐stage OA since the key genes and molecular mechanisms are unclear. Therefore, there is an urgent need to decipher the pathogenesis of OA and identify effective targets.^10^, ^11^, ^12^

Circular RNAs (circRNAs) are novel endogenous noncoding RNAs (ncRNAs) generated via alternative back‐splicing. They are much more stable than linear mRNAs due to their covalently closed loop configuration without a free 3' or 5' end.^13^ They play important roles in biological processes such as apoptosis, proliferation, and differentiation, and are involved in various human diseases, including the pathogenesis of OA.^14^, ^15^, ^16^ Nevertheless, the pathophysiologic role of circRNAs in OA remains largely unexplored. Moreover, some circRNAs are aberrantly expressed in OA cartilage, the mechanism of which remains poorly investigated.

N^6^‐methyladenosine (m6A) is a prevalent reversible RNA methylation associated with mRNAs and ncRNAs with the RRACH (R = G or A; H = A, C, or U) consensus sequence preferentially occurring around stop codons, within long exons and at 3' untranslated regions (3' UTRs).^17^, ^18^ M6A regulates their splicing, translation, export, and stability.^19^ The methylation is added and removed by methyltransferase (‘writers’) such as METTL3 and METTL14, and demethylase (‘erasers’) such as FTO and ALKBH5, respectively.^20^, ^21^ The downstream biological effects of m6A modification mainly depends on the specific binding of m6A readers such as YTH domain‐containing proteins (YTHDFs).^18^ M6A modification regulates gene expression, cell fate and involves the pathogenesis of many human diseases, including cancers, nervous system diseases, heart failure, and diabetes.^22^, ^23^, ^24^, ^25^ Notably, m6A‐modified circRNAs are endoribonuclease‐cleaved by the YTHDF2‐HRSP12‐RNase P/MRP axis.^26^ However, the role of m6A modification in the occurrence and development of OA remains largely unknown, although studies indicated the involvement of METTL3.^27^, ^28^ And the functions of m6A‐containing circRNAs in OA need to be explored.

Interferon Regulatory Factor 2 Binding Protein Like (IRF2BPL) is an E3 ubiquitin protein ligase which has been shown to drive ubiquitination and degradation of β‐catenin, thus probably inhibiting Wnt/β‐catenin signalling. IRF2BPL belongs to the IRF2BP family, which also includes IRF2BP1, IRF2BP2.^29^, ^30^ Increased β‐catenin expression is observed in OA cartilage inducing hypertrophic differentiation of chondrocytes.^31^ Wnt/β‐catenin signalling is activated during OA pathological process and inhibition of Wnt/β‐catenin signalling in mice exhibits disease‐modifying effects on DMM‐induced OA.^32^ Balanced regulation of Wnt/β‐catenin signalling in chondrocytes is essential for cartilage homeostasis.^9^ But the upstream mechanism by which Wnt/β‐catenin signalling is regulated during the pathogenesis of OA still remains largely unknown. CircRNA participates in the regulation of β‐catenin signalling in cancers, such as papillary thyroid cancer and hepatocellular carcinoma.^33^, ^34^ However, up to date, it remains unclear whether there is a functional link between circRNA and β‐catenin signalling in OA.

Here, using circRERE as an example, we highlighted the role of m6A‐modified circRNA in OA. We found that circRERE was down‐regulated in human OA cartilage and chondrocytes. In vitro and in vivo experiments revealed the chondro‐protective roles of circRERE in OA. What's more, circRERE downregulation in human OA chondrocytes is likely attributed to its increased m6A modification prone to endoribonucleolytic cleavage by YTHDF2‐HRSP12‐RNase P/MRP.

## 2 EXPERIMENTAL PROCEDURES

Experimental procedures are described in the supplementary materials and methods.

## 3 RESULTS

### 3.1 Decreased expression of circRERE/circRere in OA cartilage

**FIGURE 1:**
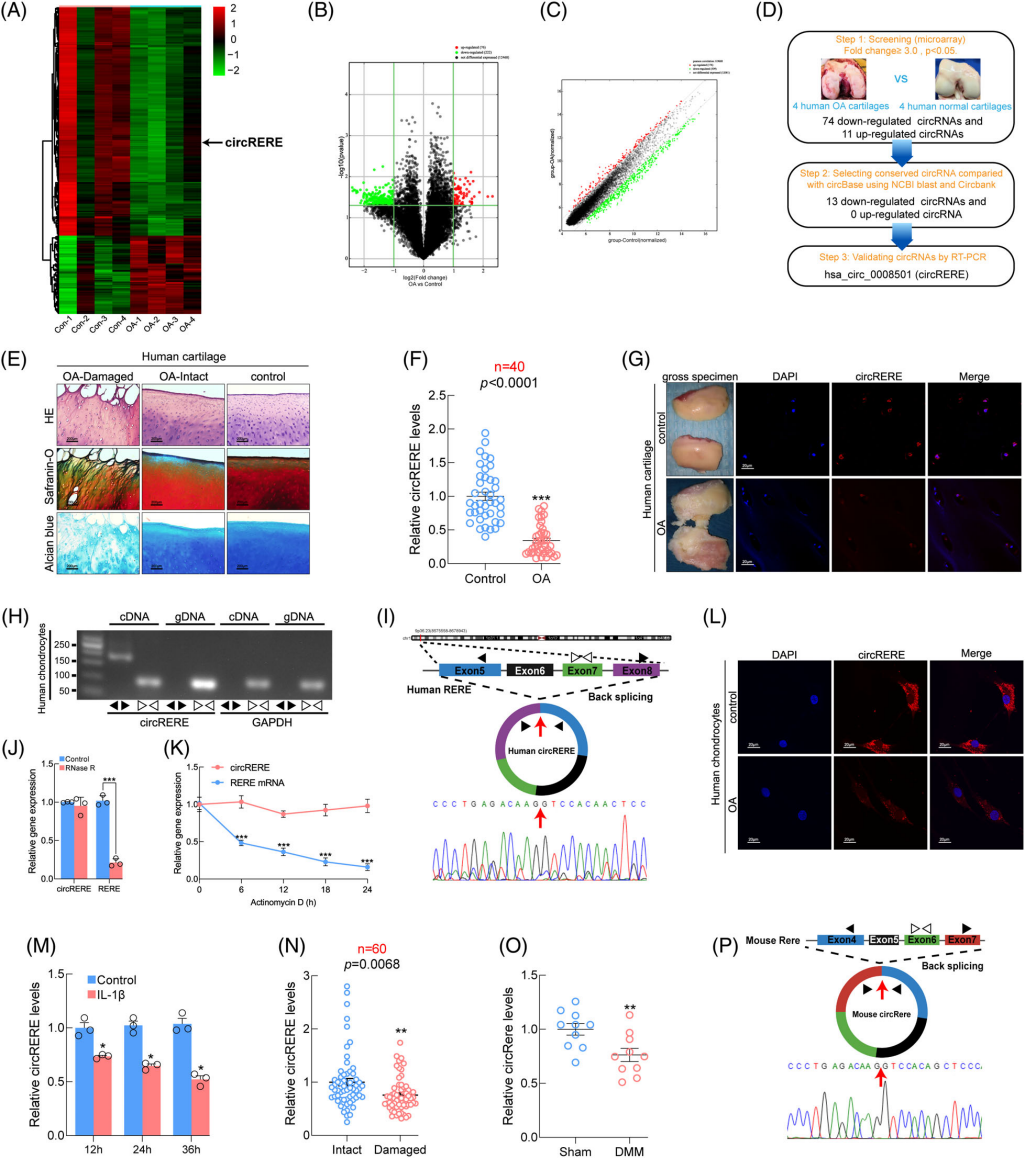
Identification of circRERE in human chondrocytes and cartilage. (A) Heat map representing differentially expressed circRNAs between human control and osteoarthritis (OA) cartilage (Fold change≥2, *p* < 0.05). CircRERE is indicated. (B, C**)** Volcano and Scatter plots illustrating the statistical significance of differentially expressed circRNAs. (D) Steps for identification of circRERE. (E) Representative histomorphological staining of human damaged and intact OA cartilage and control cartilage. Scale bar, 200 μm. (F) QRT‐PCR for expression of circRERE in human control and OA cartilage (*n* = 40). ***p < 0.01 by Mann–Whitney U test. (G) fluorescence in situ hybridization (FISH) of circRERE in human control and OA cartilage. Scale bar, 20 μm. (H, I) Validation of circRERE in HCs by RT‐PCR and Sanger sequence. (J) QRT‐PCR for the expression of circRERE and *RERE* in HCs treated with or without RNase R (*n* = 3). ***p < 0.001 by two‐tailed unpaired *t* test. (K) QRT‐PCR for the abundance of circRERE and *RERE* in HCs treated with Actinomycin D at indicated time (*n* = 3). ***p < 0.001 by two‐way ANOVA with Tukey's post hoc test. (L) FISH of circRERE in human control and OA chondrocytes. Scale bar, 20 μm. (M) Changes of circRERE in IL‐1β‐stimulated (10 ng/mL) HCs at indicated time. *p < 0.05 versus control by two‐way ANOVA with Tukey's post hoc test. (N) Relative expression of circRERE in human paired damaged and intact OA cartilage (*n* = 60). **p < 0.01 by two‐tailed Wilcoxon matched‐pairs signed rank test. (O) QRT‐PCR for expression of circRere in mouse cartilage from DMM and sham groups (*n* = 10). **p < 0.01 by two‐tailed unpaired *t* test. (P) Validation of circRere by Sanger sequence. Data are presented as mean ± SEM

The potential roles of circRNAs in OA were initially investigated using circRNA microarray of cartilage from OA patients (*n* = 4) and amputees without OA (*n* = 4) (GEO accession: GSE178724). The heat map showed 298 differentially expressed circRNAs by at least 2.0‐fold (*p* < 0.05) (Figure 1A), and the variation in expression was shown in scatter and volcano plots (Figure 1B,C). Figure 1D showed the steps for identification of circRERE. Representative histomorphological staining of human damaged and intact OA cartilage and control cartilage was shown in Figure 1E. Thirteen conserved downregulated circRNAs (at least 3.0‐fold, *p* < 0.05) were identified between humans and mice using the circbank database.^35^ Five circRNAs were non‐specifically amplified (circFMN2, circTLK1, circDENND1A, circSMARCB1, circRNF114), three were undetected (circLDLRAD4, circRMDN2, circFRAS1), and five were further detected between human OA and control cartilage using quantitative RT‐PCR (qRT‐PCR) (*n* = 20) (Figure S1A, B). QRT‐PCR showed that the expression levels of CircZFHX4, circTBCK, circARHGAP5 and circRERE significantly decreased in OA cartilage compared with control, with no significant difference in circTENM3. Because circRERE had the greatest difference in expression, its transcription was confirmed in the validation set (*n* = 40) (Figure 1F), and the differential expression was confirmed by fluorescence in situ hybridization (FISH) (Figure 1G), but differential *RERE* expression was not observed (Figure S1B). Thus, circRERE was selected for further study. The spliced mature circRERE sequence (434 bp) was derived from exons 5–8 of the gene RERE (Figure 1H,I). CircRERE was more stable than linear *RERE* determined by RNase R digestion and actinomycin D treatment (Figure 1J,K). FISH of human chondrocytes showed that circRERE was predominantly located in the cytoplasm and decreased in OA chondrocytes compared with control (Figure 1L). Downregulated circRERE was also found in IL‐1β‐treated HCs in a time‐dependent manner (Figure 1M). Furthermore, downregulated circRERE expression was observed in damaged OA cartilage compared with intact cartilage from the same patient (*n* = 60) (Figure 1N). The mouse homologue, circRere was also examined due to the conservation of circRERE between humans and mice (Figure S1C). Decreased circRere expression was observed in the cartilage of mice subjected to the surgery of destabilization of medial meniscus (DMM) compared with that of sham group (Figure 1O). Circularisation, stability, and location of circRere were validated in mouse chondrocytes (MCs) (Figure 1P and Figure S1D‐F). These results indicated that circRERE/circRere may exert disease‐specific effects in the pathogenesis of OA.

### 3.2 Overexpression of circRERE (circRere) ameliorated osteoarthritis in vitro (in vivo*)*

**FIGURE 2:**
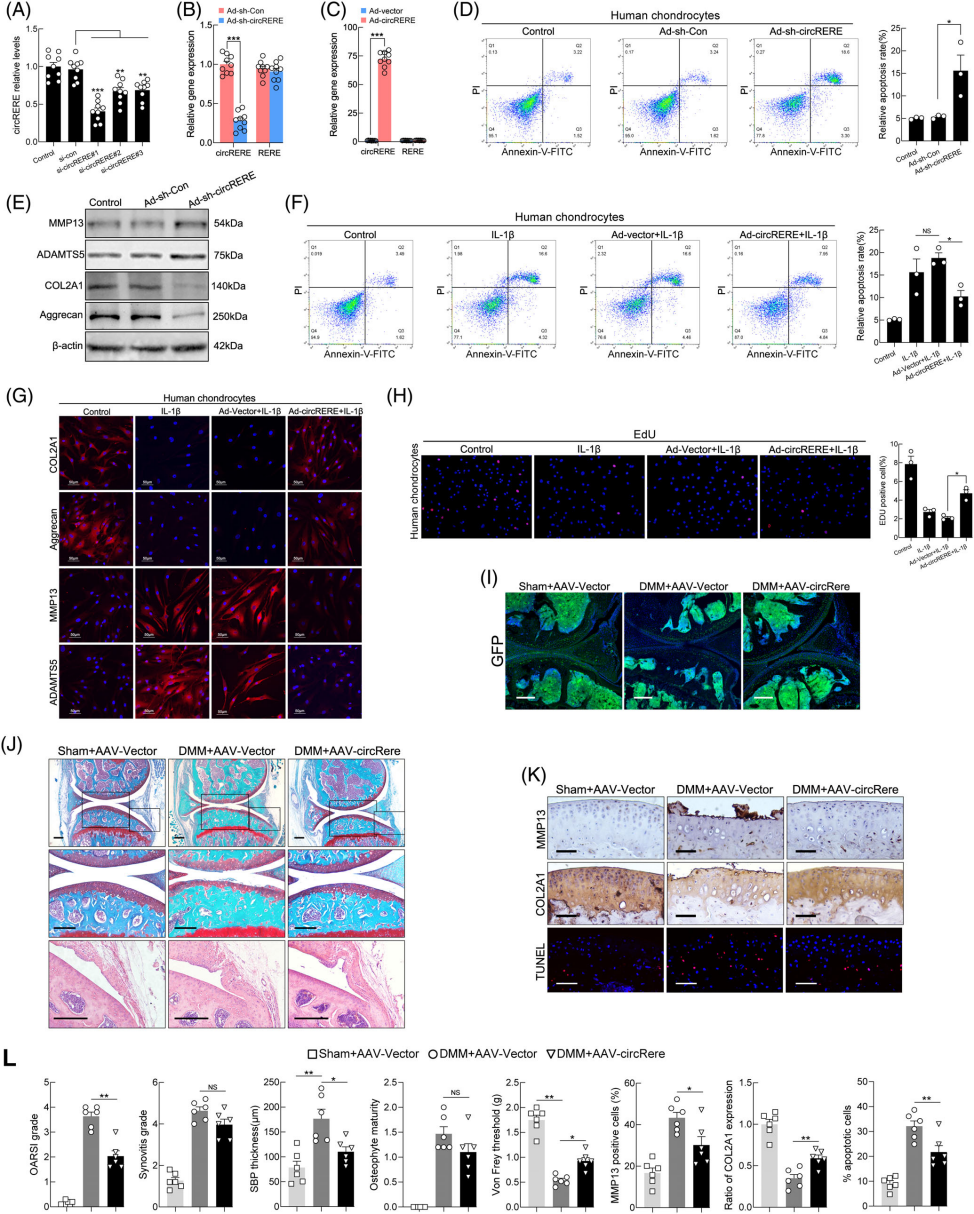
Overexpression of circRERE/circRere ameliorated osteoarthritis (OA) progression. (A) QRT‐PCR for circRERE expression in HCs transfected with circRERE siRNA or negative control (*n* = 9, concentration of 20 nM, 48 h). **p < 0.01, ***p < 0.001 by one‐way ANOVA with Tukey's post hoc test. (B, C) QRT‐PCR for circRERE and *RERE* expression in HCs infected with Ad‐sh‐Con, Ad‐sh‐circRERE, Ad‐vector or Ad‐circRERE (*n* = 9). ***p < 0.001 by two‐tailed unpaired *t* test. (D, E) HCs were infected with Ad‐sh‐Con or Ad‐sh‐circRERE. Cell apoptosis was determined by flow cytometry (FCM) (D, *n* = 3). *p < 0.05 by one‐way ANOVA with Tukey's post hoc test. Western blotting (WB) analysis for MMP13, ADAMTS5, COL2A1 and Aggrecan in HCs (E). (F‐H) HCs were infected with Ad‐vector or Ad‐circRERE and stimulated with IL‐1β (10 ng/mL) for 48 h. FCM assays for detection of apoptosis in HCs (*n* = 3, F). *p < 0.05 by one‐way ANOVA with Tukey's post hoc test. Immunofluorescence (IF) of COL2A1, Aggrecan, MMP13 and ADAMTS5 in HCs (G). Scale bar, 50 μm. Proliferation of HCs was determined by EdU assays (*n* = 3, H). *p < 0.05 by one‐way ANOVA with Tukey's post hoc test. (I) To investigate the infected efficiency of AAV, representative knee cartilage fluorescence (GFP) images in knee sections from three groups were obtained by a confocal microscope. Scale bar, 200 μm. (J) Representative images of Safranin O‐fast green and haematoxylin–eosin (HE) staining in knee sections from three groups 8 weeks after DMM. Scale bar, 200 μm. (K) IHC staining for MMP13 and COL2A1, and TUNEL assay in mouse cartilage. Scale bar, 50 μm. (L) Scoring of OA parameters (OARSI grade, synovitis score, subchondral bone plate (SBP) thickness, osteophyte maturity and Von Frey threshold). Quantification of MMP13 and COL2A1 expression, and apoptotic chondrocytes in mouse cartilage (*n* = 6). *p < 0.05, **p < 0.01 by one‐way ANOVA with Tukey's post hoc test. Data are presented as mean ± SEM. NS, no significance

For circRERE interference, three different siRNAs were designed and tested in HCs (Figure 2A). The role of circRERE in OA in vitro was examined by circRERE downregulation or overexpression in HCs using adenovirus expressing shRNA#1 against circRERE (Ad‐sh‐circRERE) or adenovirus overexpressing circRERE (Ad‐circRERE). *RERE* expression was unaffected by circRERE downregulation or overexpression (Figure 2B,C). CircRERE silencing increased apoptotic HCs and affected the protein levels of MMP13, ADAMTS5, COL2A1 and Aggrecan, whereas its overexpression reversed IL‐1β‐induced apoptosis of HCs (Figure 2D‐F). In addition, the chondroprotective effect of circRERE overexpression on HCs stimulated with IL‐1β was supported by immunofluorescence (IF) and EdU assays (Figure 2G,H). However, this effect was unseen by senescence‐associated‐β‐galactosidase (β‐gal) staining (Figure S2A).

Due to the conservation of circRERE and circRere between humans and mice, the role of circRere in OA progression in vivo was investigated using the surgery of DMM on 10‐week‐old male C57BL/6 mice and AAV infection. An AAV system has been demonstrated to effectively deliver target genes to cartilage and other joint tissues.^36^, ^37^ GFP‐tagged AAV‐circRere or AAV‐vector was intra‐articular (IA) injected into the affected knees of DMM mice (commencing 1 week after DMM surgery) (Figure S2B). Infected efficiency was determined by fluorescence examination of knee sections and qRT‐PCR of cartilage (Figure 2I and Figure S2C). DMM + AAV‐circRere group exhibited reductions in the examined manifestations of OA compared with DMM + AAV‐vector group 8 weeks after DMM, including cartilage destruction, subchondral bone plate (SBP) thickening and pain, but not synovitis and osteophyte formation (Figure 2J,L). In addition, immunohistochemical (IHC) staining and TUNEL assay showed that the DMM‐induced upregulation of MMP13 proteins, downregulation of COL2A1 proteins and upregulation of apoptotic chondrocytes in damaged mouse cartilage were significantly diminished in DMM + AAV‐circRere group (Figure 2K,L), which further revealed the chondroprotective effects of circRere in vivo. These results indicated that circRERE/circRere had important roles in pathophysiologic process of OA.

### 3.3 M6A‐modification of circRERE modulated its expression and function in OA chondrocytes

Next, the mechanism underlying circRERE downregulation in OA chondrocytes was explored. M6A not only regulates the generation, transport, stability and degradation of targeted genes (such as mRNA, circRNA), but also influences the corresponding biological function and processes by affecting the targeted gene expression.^38^, ^39^ Particularly, m6A‐modified circRNAs are reported to be endoribonuclease‐cleaved by the axis of YTHDF2‐HRSP12‐RNase P/MRP.^26^

**FIGURE 3:**
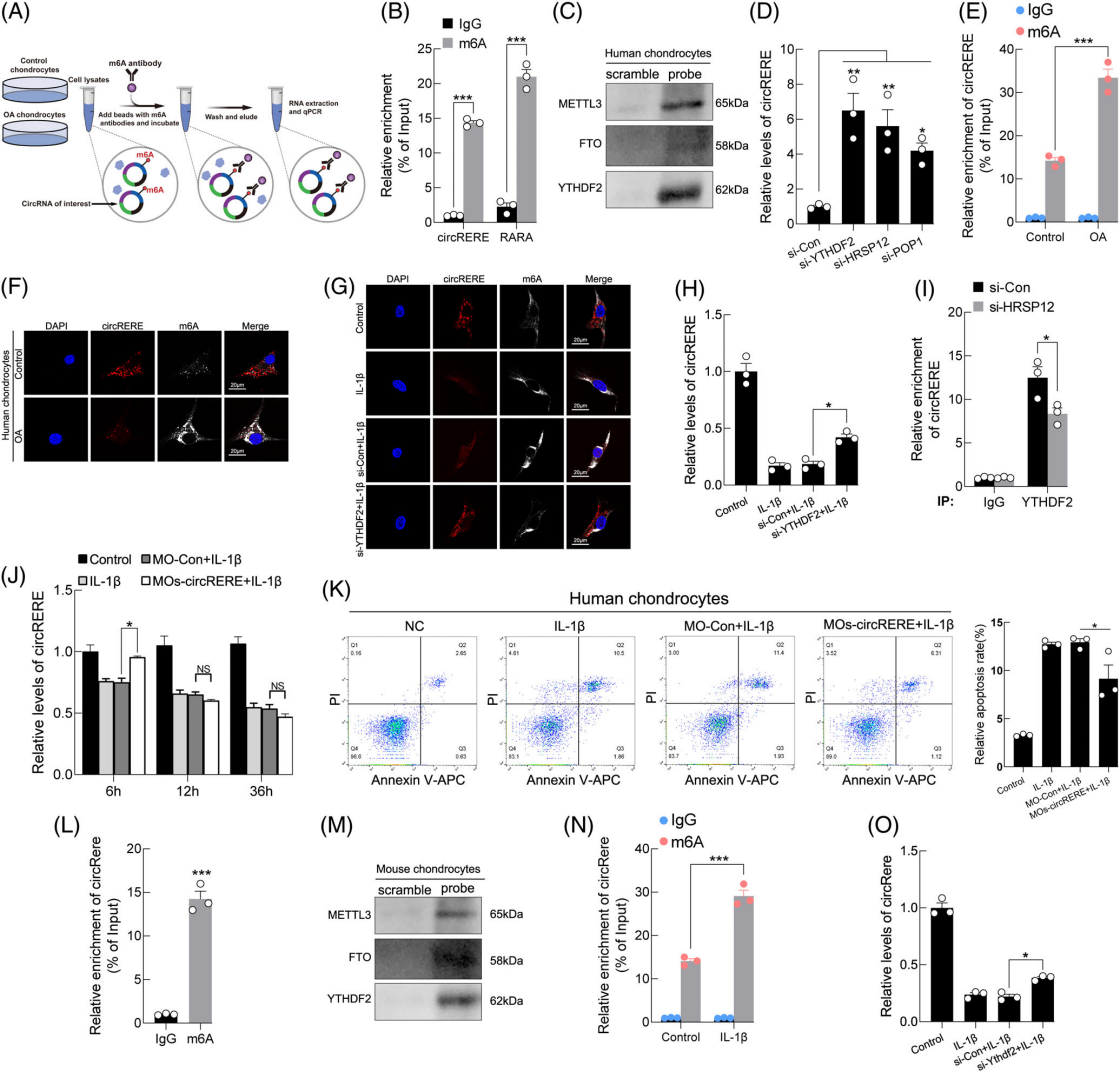
Modulation of m6A methylation on circRERE. (A) Flow chart of m6A‐specific immunoprecipitation (MeRIP) assays. (B) MeRIP assay showing that circRERE was highly enriched in immunoprecipitates (IPs) of m6A antibody (*n* = 3). ***p < 0.001 by two‐tailed unpaired *t* test. *RARA*, which is a known m6A‐containing RNA, was used as a positive control. (C) RNA pulldown assays and WB analysis for METTL3, FTO and YTHDF2 using a circRERE probe. (D) The expression of endogenous circRERE in HCs transfected with indicated siRNA (at final concentration of 20 nM) (*n* = 3). *p < 0.05, **p < 0.01 by one‐way ANOVA with Tukey's post hoc test. (E) MeRIP assays indicating the increased m6A‐modification of circRERE in human osteoarthritis (OA) chondrocytes compared with control (*n* = 3). The percentage of the input is shown. ***p < 0.001 by two‐way ANOVA with Tukey's post hoc test. (F) Fluorescence in situ hybridization (FISH) (circRERE)‐IF (m6A) staining in human OA and control chondrocytes. (G, H) FISH (circRERE)‐IF (m6A) staining (G) and qRT‐PCR for the expression of circRERE in HCs transfected with si‐YTHDF2 and stimulated with IL‐1β (10 ng/mL) for 48 h (H, *n* = 3). *p < 0.05 by one‐way ANOVA with Tukey's post hoc test. (I) CO‐IP of YTHDF2 in HCs transfected with si‐Con or si‐HRSP12 (*n* = 3). The amount of CO‐IPed endogenous circRERE was normalized to the level of endogenous GAPDH mRNA. Then, the normalized levels obtained in IPs with IgG in si‐Con transfected HCs were arbitrarily set to 1. *p < 0.05 by two‐tailed unpaired *t* test. (J) QRT‐PCR for circRERE expression in HCs transfected with MOs‐circRERE and stimulated with IL‐1β (10 ng/mL) at indicated time (*n* = 3). *p < 0.05 by two‐way ANOVA with Tukey's post hoc test. (K) FCM of HCs treated with MOs‐circRERE and stimulated with IL‐1β for 6 h (*n* = 3). *p < 0.05 by one‐way ANOVA with Tukey's post hoc test. (L) MeRIP assay showing that circRere was highly enriched in IPs of m6A antibody (*n* = 3). ***p < 0.001by two‐tailed unpaired *t* test. (M) RNA pulldown assays and WB analysis for METTL3, FTO and YTHDF2 using a circRere probe. (N) MeRIP assay showing the increased percentage of m6A‐containing circRere in MCs treated with IL‐1β (10 ng/mL, 48 h) compared with control (*n* = 3). ***p < 0.001 by two‐way ANOVA with Tukey's post hoc test. (O) QRT‐PCR for circRere expression in MCs transfected with si‐Ythdf2 and stimulated with IL‐1β. *p < 0.05 by one‐way ANOVA with Tukey's post hoc test. Data are presented as mean ± SEM

To investigate the effects of m6A modification on circRERE expression or function, we first examined whether circRERE contains m6A methylation, m6A‐specific immunoprecipitation (MeRIP) assays and subsequent qRT‐PCR analysis using divergent primers showed that circRERE was enriched in m6A antibody‐precipitated complexes (Figure 3A,B). RNA pulldown assays revealed that circRERE interacted with several m6A modifiers: METTL3, FTO, and YTHDF2 (Figure 3C). Thus, we hypothesized that m6A‐containing circRERE was subjected to endoribonucleolytic cleavage by YTHDF2‐HRSP12‐RNase P/MRP. Downregulation of YTHDF2, HRSP12, or POP1 in HCs increased the abundance of circRERE, which confirmed above hypothesis (Figure 3D). Furthermore, the percentage of m6A‐modified circRERE was upregulated in human OA chondrocytes compared with control observed by MeRIP assays and IF (m6A)‐FISH (circRERE) staining (Figure 3E,F). Above results indicate the possibility that increased m6A modification of circRERE in OA chondrocytes enhances its endoribonucleolytic degradation by YTHDF2‐HRSP12‐RNase P/MRP, and this was supported by the following experiments.

IF (m6A)‐FISH (circRERE) staining and qRT‐PCR assays suggested that decreased circRERE expression in response to IL‐1β stimulation was partly abrogated by the downregulation of YTHDF2 in HCs (Figure 3G,H). Given the evidence that m6A‐containing circRNAs which are associated with YTHDF2 in a pattern of HRSP12‐dependence are preferentially subjected to endoribonucleolytic cleavage by RNase P/MRP.^26^ Here, Figure 3I showed the observed enrichment of YTHDF2 with circRERE was obviously reversed by HRSP12 downregulation. Thus, morpholino oligos (MOs) targeting two HRSP12‐binding motifs (GGUUC) in circRERE (MOs‐circRERE) were designed by browsing its sequence.^40^, ^41^ Transfection with MOs‐circRERE partially reversed the decreased expression of circRERE in HCs stimulated with IL‐1β for 6 h, but not 12 or 36 h (Figure 3J). Notably, flow cytometry (FCM) assay showed that increased apoptosis of HCs upon 6 h IL‐1β treatment was partly rescued by transfection with MOs‐circRERE (Figure 3K). Conserved mouse circRere also contains m6A methylation (Figure 3L,M), and the percentage of m6A‐modified circRere was upregulated in IL‐1β‐treated MCs compared with control (Figure 3N). And decreased circRere expression in response to IL‐1β stimulation was partly rescued by the downregulation of Ythdf2 in MCs (Figure 3O). Together, all results suggested that increased m6A modification of circRERE in OA chondrocytes, through recognition by YTHDF2‐HRSP12‐RNase P/MRP, modulated its degradation and function.

### 3.4 Restoration of circRERE expression alleviated OA by targeting miR‐195‐5p

**FIGURE 4:**
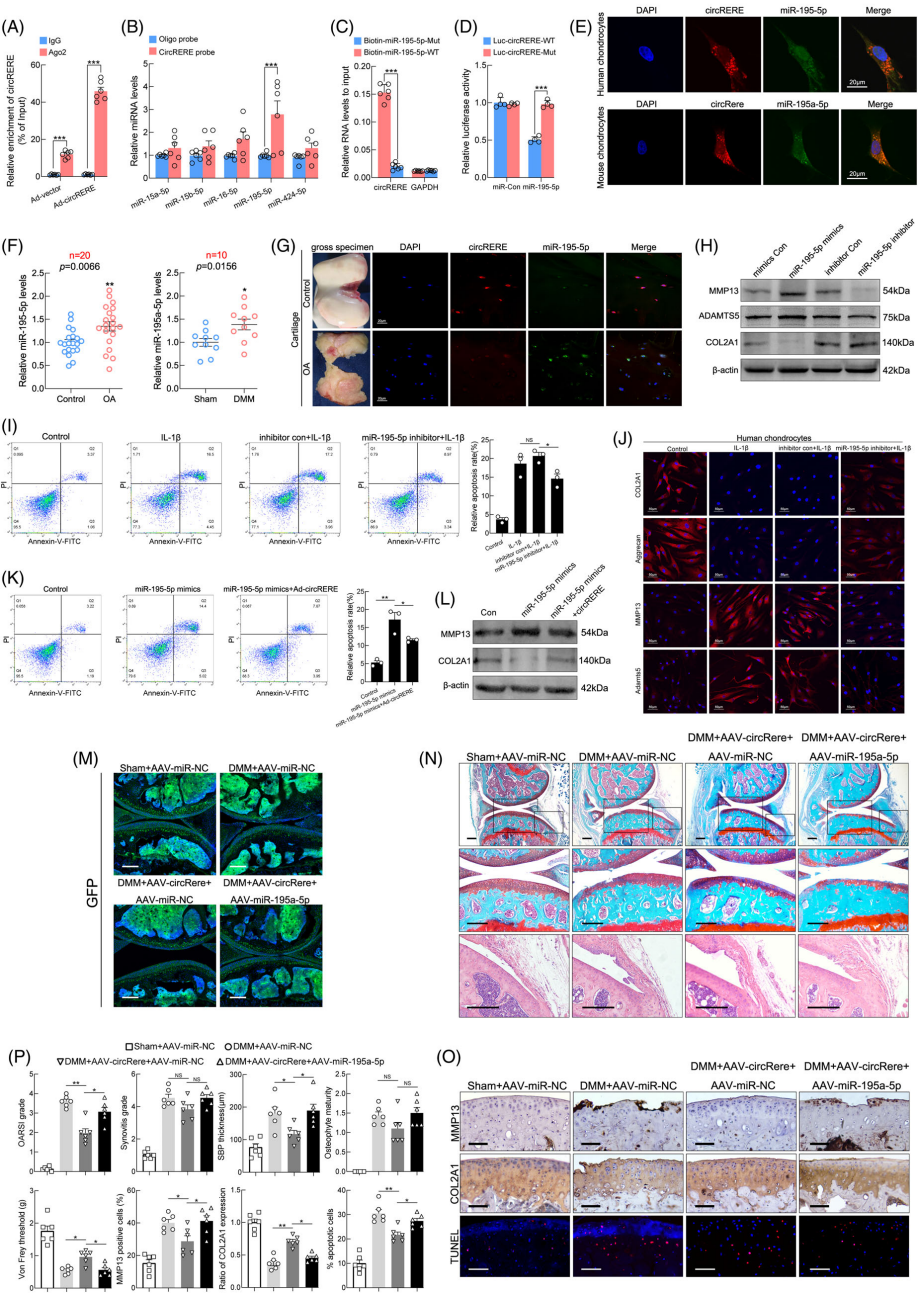
CircRERE functions as a sponge for miR‐195‐5p in osteoarthritis (OA). (A) Ago2 RIP assay was performed to detect circRERE levels in HCs infected with Ad‐vector or Ad‐circRERE (*n* = 6). ***p < 0.001 by two‐tailed unpaired Welch's *t* test. (B) RNA pull‐down and qRT‐PCR assays for miRNAs in HCs lysates pull‐downed by circRERE or oligo probe (*n* = 6). ***p < 0.001 by two‐tailed unpaired Welch's *t* test. (C) Wild‐type (WT) or mutant (Mut) biotinylated‐miR‐195‐5p mimics were transfected into circRERE overexpressing HCs. After streptavidin capture, circRERE levels were analysed by qRT‐PCR (*n* = 6). ***p < 0.001 by two‐tailed unpaired *t* test. (D) MiR‐195‐5p mimic or control was co‐transfected with WT or MUT circRERE luciferase reporter vector into HEK293T cells (*n* = 4). ***p < 0.001by two‐tailed unpaired *t* test. (E) Fluorescence in situ hybridization (FISH) of circRERE/miR‐195‐5p and circRere/miR‐195a‐5p in HCs and MCs. (F) QRT‐PCR for miR‐195‐5p in human OA and control cartilage (Left, *n* = 20). **p < 0.01 by two‐tailed unpaired Welch's *t* test. QRT‐PCR for miR‐195a‐5p in mouse cartilage from DMM and sham groups (Right, *n* = 10). *p < 0.05 by two‐tailed unpaired *t* test. (G) FISH of circRERE/miR‐195‐5p in human control and OA cartilage. (H) WB analysis for MMP13, ADAMTS5 and COL2A1 in HCs upon different transfections. (I, J) HCs were transfected with miR‐195‐5p inhibitor or control and stimulated with IL‐1β (10 ng/mL, 48 h). Cell apoptosis was determined by FCM (I, *n* = 3). *p < 0.05 by one‐way ANOVA with Tukey's post hoc test. IF of COL2A1, Aggrecan, MMP13 and ADAMTS5 in HCs (J). (K) Overexpression of both miR‐195‐5p and circRERE resulted in decreased apoptosis of HCs compared with HCs overexpressing miR‐195‐5p alone (*n* = 3). *p < 0.05 by one‐way ANOVA with Tukey's post hoc test. (L) WB analysis for MMP13 and COL2A1 in HCs under the same experimental condition of Figure 4K. (M) To investigate the infected efficiency of AAVs, representative knee cartilage fluorescence (GFP) images in knee sections from four groups were obtained by a confocal microscope. Scale bar, 200 μm. (N) Representative images of Safranin O‐fast green, HE staining in knee sections from four groups 8 weeks after DMM. Scale bar, 200 μm. (O) IHC staining for MMP13 and COL2A1, and TUNEL assay in mouse cartilage. Scale bar, 50 μm. (P) Scoring of OA parameters. Quantification of MMP13 and COL2A1 expression, and apoptotic chondrocytes in mouse cartilage (*n* = 6). *p < 0.05, **p < 0.01 by one‐way ANOVA with Tukey's post hoc test. Data are presented as mean ± SEM

The cytoplasmic localization of circRERE indicated its potential molecular mechanism in regulating OA may be microRNA (miRNA) sponging, peptide encoding, or circRNA‐protein interactions.^42^ The Circular RNA Interactome database predicted that AGO2, IGF2BP3, FMRP, EIF4A3, and DGCR8 binds to circRERE (Figure S4A).^43^ RIP assays showed that circRERE interacted with AGO2, FMRP, and IGF2BP3, with AGO2 being the most abundant (Figure 4A and Figure S4B). Therefore, we focused on the ability of circRERE to function as a competing endogenous RNA (ceRNA).^44^, ^45^, ^46^

CircRERE may bind miR‐16‐5p, miR‐424‐5p, miR‐15a‐5p, miR‐15b‐5p, and miR‐195‐5p according to the overlapping predictions of miRanda, TargetScan, and Arraystar's proprietary program predicted miRNA (Figure S4C). RNA pull‐down and qRT‐PCR assays showed that miR‐195‐5p was abundantly pulled down by circRERE in HCs (Figure 4B). The direct interaction between circRERE and miR‐195‐5p was confirmed since the biotinylated wild‐type miR‐195‐5p mimics captured more circRERE compared to mutant miR‐195‐5p in circRERE‐overexpressing HCs (Figure 4C). Furthermore, luciferase reporter assays confirmed the binding of circRERE to miR‐195‐5p (Figure 4D and Figure S4F). FISH revealed the co‐localization of circRERE/circRere and miR‐195‐5p in HCs/MCs (Figure 4E). Therefore, miR‐195‐5p was chosen for further study. QRT‐PCR indicated that miR‐195‐5p expression was higher in OA cartilage than in control (*n* = 20 for human, *n* = 10 for mice) (Figure 4F). Differential expression of circRERE and miR‐195‐5p were confirmed in human OA and control cartilage by FISH (Figure 4G).

The functions of miR‐195‐5p in vitro were investigated using FCM, EdU, and IF assays. Loss‐of‐function experiments suggested that downregulation of miR‐195‐5p in HCs rescued IL‐1β‐induced apoptosis and aberrant expression of anabolic and catabolic molecules (Figure 4I,J), but did not affect proliferation (Figure S4J,K). The effect of miR‐195‐5p overexpression or downregulation on the protein levels of MMP13, ADAMTS5 and COL2A1 in HCs was confirmed by western blotting (WB) (Figure 4H and Figure S4I). Furthermore, increased apoptosis of HCs in miR‐195‐5p overexpression group was counteracted by circRERE overexpression (Figure 4K), indicating that circRERE is involved in OA by sponging miR‐195‐5p. This mechanism was supported by WB (Figure 4L and Figure S4L).

Considering that circRERE is conserved between humans and mice, and nucleotides upstream and downstream of the miR‐195‐5p target sequences in circRERE and circRere were also highly conserved (Figure S4D,E). We further investigated whether circRere is involved in OA in mice by targeting miR‐195a‐5p. GFP‐tagged AAV‐circRere and AAV‐miR‐195a‐5p were co‐IA‐injected into the operated knees in DMM mice (commencing 1 week after DMM surgery) as Figure S2B and the infected efficiency was confirmed by fluorescence examination of knee sections and qRT‐PCR analysis of cartilage (Figure 4M and Figure S4M).

Marked increases in the examined manifestations of DMM‐induced OA were observed in the DMM + AAV‐miR‐NC and DMM + AAV‐circRere + AAV‐miR‐195a‐5p groups compared with the Sham+AAV‐miR‐NC and DMM + AAV‐circRere + AAV‐miR‐NC groups, including cartilage erosion, SBP thickness and pain, but not synovitis and osteophyte maturation (Figure 4N,P), indicating that the protective effects of AAV‐circRere on DMM‐induced OA were counteracted by AAV‐miR‐195a‐5p overexpression in mice. Furthermore, IHC staining for MMP13 and COL2A1 and TUNEL assays in mouse cartilage of four groups were consistent with above results of Safranin O‐fast green and HE staining and Von Frey assays (Figure 4O,P).

### 3.5 CircRERE modulated β‐catenin ubiquitination and degradation by targeting miR‐195‐5p/IRF2BPL in human chondrocytes

**FIGURE 5:**
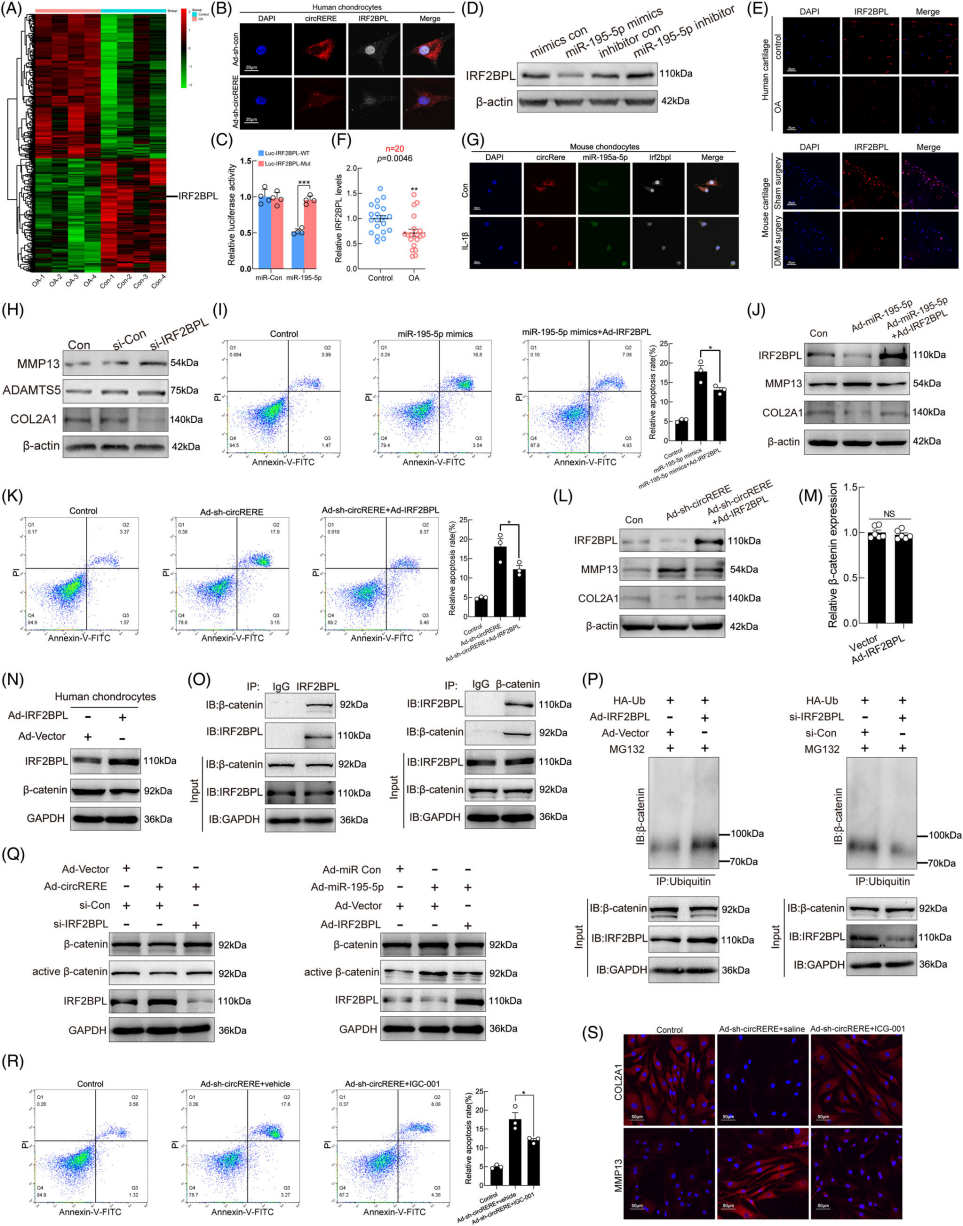
Modulation of circRERE on β‐Catenin ubiquitination and degradation via targeting miR‐195‐5p/IRF2BPL in human chondrocytes. (A) Heat map representing all differentially expressed mRNAs between human osteoarthritis (OA) and control cartilage (Fold change≥2, *p* < 0.05). IRF2BPL is indicated. (B) IF (IRF2BPL)‐fluorescence in situ hybridization (FISH) (circRERE) assay showing the decreased IRF2BPL protein level upon circRERE knockdown in HCs. Scale bar, 20 μm. (C) MiR‐195‐5p mimic or mimic control was co‐transfected with WT or MUT IRF2BPL 3'‐UTR luciferase reporter vector into HEK‐293 T cells (*n* = 4). ***p < 0.001by two‐tailed unpaired *t* test. (D) WB analysis for IRF2BPL in HCs upon different transfections. (E) IF of IRF2BPL in control and OA cartilage from humans and mice. Scale bar, 20 μm. (F) QRT‐PCR for *IRF2BPL* expression in human OA and control cartilage (*n* = 20). **p < 0.01 by Mann–Whitney U test. (G) FISH‐IF staining of circRere/miR‐195a‐5p/IRF2BPL in MCs treated with IL‐β (10 ng/mL, 48 h) or not. Scale bar, 20 μm. (H) Related protein levels in HCs. (I) Overexpression of both miR‐195‐5p and IRF2BPL resulted in decreased apoptosis of HCs compared with HCs overexpressing miR‐195‐5p alone (*n* = 3). *p < 0.05 by one‐way ANOVA with Tukey's post hoc test. (J) Related protein levels of HCs under the same experimental conditions of Figure 5I. (K) CircRERE knockdown with IRF2BPL overexpression resulted in fewer apoptotic HCs than those observed with circRERE knockdown alone (*n* = 3). *p < 0.05 by one‐way ANOVA with Tukey's post hoc test. (L) WB analysis for related proteins in HCs. (M, N) HCs were infected with Ad‐Vector or Ad‐IRF2BPL. Then mRNA and protein levels of β‐catenin were detected by qRT‐PCR (M) and WB (N) respectively. (O) CO‐IP of IRF2BPL and β‐catenin in HCs. (P) β‐catenin ubiquitination level upon IRF2BPL overexpression or downregulation was detected by IP. HCs were incubated with indicated adenoviruses, plasmid and siRNA. (Q) WB analysis for β‐catenin, active β‐catenin, IRF2BPL, and GAPDH in HCs after incubation with indicated adenoviruses and siRNA. (R) CircRERE downregulation with ICG‐001 treatment (5 μM) resulted in decreased apoptotic HCs compared with those observed with circRERE downregulation alone (*n* = 3). *p < 0.05 by one‐way ANOVA with Tukey's post hoc test. (S) IF of COL2A1 and MMP13 in HCs. Data are presented as mean ± SEM

We then investigated the ability of circRERE to exert a chondroprotective role by modulating expression of miR‐195‐5p targeting genes. An mRNA microarray (GEO accession: GSE178557) was performed using the same cartilage samples as the aforementioned circRNA microarray (at least 2.0‐fold, *p* < 0.05, Figure 5A and Figure S5A,B). And Gene Ontology (GO) analysis revealed that downregulated mRNAs in OA cartilage were involved in extracellular matrix structural constituent (GO:0005201) and extracellular matrix (GO:0031012), and upregulated mRNAs in OA cartilage were enriched in cell death (GO:0008219), cellular catabolic process (GO:0044248), programmed cell death (GO:0012501), and apoptotic process (GO:0006915) (Figure S5C‐H). Integration of mRNA microarray data and predictions using PITA, miRanda, Pictar, and TargetScan indicated nine possible targets of miR‐195‐5p (Figure S5K). QRT‐PCR suggested that interferon regulatory factor 2 binding protein like (*IRF2BPL*) was modulated by circRERE silencing (Figure S5L). IF (IRF2BPL)‐FISH (circRERE) staining confirmed that downregulation of circRERE decreased IRF2BPL protein levels in HCs (Figure 5B). The TargetScan database also showed the conservation of miR‐195‐5p and its target *IRF2BPL* gene in humans and mice (Figure S5M). Luciferase reporter assays showed that miR‐195‐5p overexpression decreased the luciferase activity of WT IRF2BPL 3'‐UTR, but not the mutant (Figure 5C and Figure S5N). Furthermore, WB revealed that transfection with miR‐195‐5p mimics downregulated IRF2BPL protein levels in HCs, whereas miR‐195‐5p inhibitor displayed the opposite effect (Figure 5D). IF staining and qRT‐PCR showed that IRF2BPL expression was significantly downregulated in OA cartilage from humans and mice (Figure 5E,F and Figure S5O), and the downregulated expression of Irf2bpl was also observed in IL‐1β stimulated MCs (Figure 5G), indicating its potential roles in OA pathogenesis. Therefore, IRF2BPL was selected for further analyses. IRF2BPL downregulation affected the expression of anabolic and catabolic molecules in HCs (Figure 5H). In addition, rescue experiments suggested that IRF2BPL overexpression abolished miR‐195‐5p overexpression‐ or circRERE knockdown‐induced apoptosis and aberrant MMP13 and COL2A1 expression in HCs (Figure 5I‐L). Hence, circRERE exerted chondroprotective effects by modulating the expression of miR‐195‐5p targeting *IRF2BPL*.

IRF2BPL is an E3 ubiquitin protein ligase which can drive proteasome‐mediated ubiquitin‐dependent degradation of β‐catenin, and β‐catenin expression increases proportionally with OA severity.^30^, ^31^ Thus, we studied if the chondro‐regulatory mechanism of circRERE‐miR‐195‐5p‐IRF2BPL axis was mediated by the role of IRF2BPL in β‐catenin ubiquitination and degradation. First, overexpression of IRF2BPL in HCs did not affect β‐catenin mRNA expression (Figure 5M), but markedly decreased β‐catenin protein level (Figure 5N). Co‐immunoprecipitation (IP) in HCs revealed an interaction between IRF2BPL and β‐catenin (Figure 5O). Second, endogenous β‐catenin ubiquitination in HCs increased and decreased during IRF2BPL overexpression and knockdown, respectively (Figure 5P). Third, there was downregulated expression and suppressed activation of β‐catenin in HCs overexpressing circRERE, whereas the effects were abrogated after IRF2BPL knockdown. Similarly, the effects of miR‐195‐5p overexpression on β‐catenin expression and activation were counteracted by IRF2BPL overexpression (Figure 5Q and Figure S5P,Q). Fourth, the chondro‐destructive effects of circRERE downregulation on HCs were diminished by ICG‐001, a small molecule that antagonizes TCF/β‐catenin‐mediated transcription,^47^ determined by FCM and IF assays of HCs (Figure 5R,S). Collectively, our data indicated that circRERE participated in pathophysiologic process of OA by targeting miR‐195‐5p/IRF2BPL to regulate β‐catenin ubiquitination and degradation.

### 3.6 CircRere targets miR‐195a‐5p/Irf2bpl/β‐catenin to participate in the pathophysiologic process of mouse OA in vivo

**FIGURE 6:**
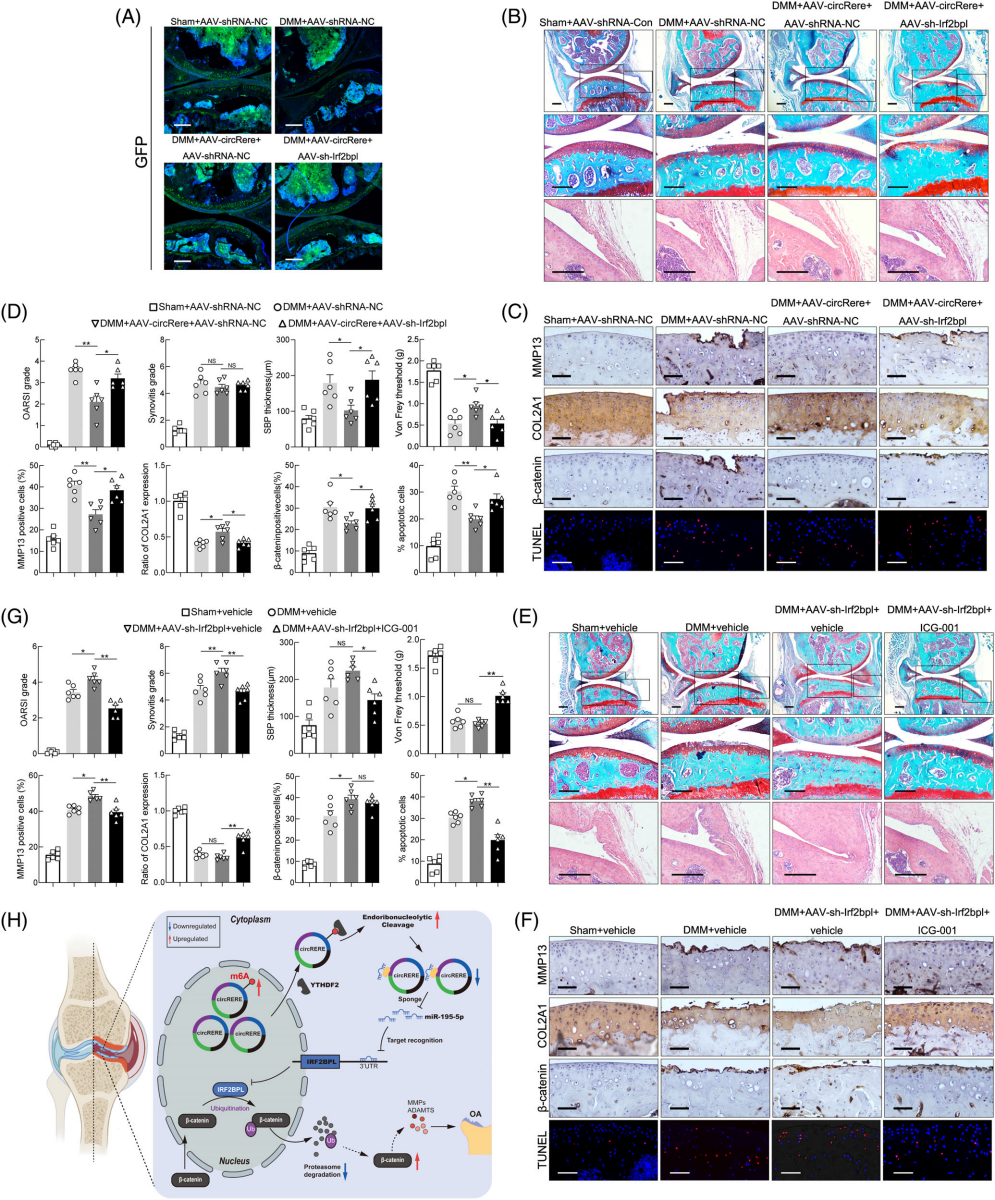
The role of circRere/miR‐195a‐5p/Irf2bpl/β‐catenin axis in the progression of DMM‐induced osteoarthritis (OA). (A) To investigate the infected efficiency of AAVs, representative knee cartilage fluorescence (GFP) images in knee sections from four groups were obtained by a confocal microscope. Scale bar, 200 μm. (B) Representative images of Safranin O‐fast green and HE staining in knee sections from four groups 8 weeks after DMM. Scale bar, 200 μm. (C) IHC staining for MMP13, COL2A1 and β‐catenin and TUNEL assay in mouse cartilage from above four groups. Scale bar, 50 μm. (D) Scoring of OA parameters. Quantification of MMP13, COL2A1 and β‐catenin expression, and apoptotic chondrocytes in mouse cartilage (*n* = 6). *p < 0.05, **p < 0.01 by one‐way ANOVA with Tukey's post hoc test. (E) Representative images of Safranin O‐fast green, HE staining in knee sections from four groups 8 weeks after DMM. Scale bar, 200 μm. (F) IHC staining for MMP13, COL2A1 and β‐catenin and TUNEL assay in mouse cartilage from four groups. Scale bar, 50 μm. (G) Scoring of OA parameters. Quantification of MMP13, COL2A1 and β‐catenin expression, and apoptotic chondrocytes in mouse cartilage (*n* = 6). *p < 0.05, **p < 0.01 by one‐way ANOVA with Tukey's post hoc test. (H) Proposed schematic of m6A‐moidified circRERE and downstream targets during the pathogenesis of OA. Data are presented as mean ± SEM

Considering the conservation of circRERE/miR‐195‐5p/IRF2BPL axis in humans and mice, the role of circRere/miR‐195a‐5p/Irf2bpl/β‐catenin axis in OA was further tested in vivo by performing DMM surgery on mice and co‐IA injection with corresponding AAVs (or antagonist). For deprivation experiments, AAV‐circRere and AAV‐sh‐Irf2bpl were co‐IA injected into the affected knees (commencing 1 week after DMM surgery). Infected efficiency was determined by fluorescence examination of knee sections and qRT‐PCR of cartilage (Figure 6A and Figure S6A).

Marked increases in the examined manifestations of DMM‐induced OA were observed in the DMM + AAV‐shRNA‐NC and DMM + AAV‐circRere + AAV‐sh‐Irf2bpl groups compared with the Sham + AAV‐shRNA‐NC and DMM + AAV‐circRere+AAV‐shRNA‐NC groups, including cartilage erosion, SBP thickness and pain, but not synovitis and osteophyte maturation (Figure 6B,D and Figure S6B), indicating that the protective effects of AAV‐circRere on OA progression caused by DMM in mice were counteracted by AAV‐sh‐Irf2bpl. In addition, IHC staining for MMP13 and COL2A1 and TUNEL assays in mouse cartilage of four groups were consistent with the above results. Particularly, the β‐catenin levels in mouse cartilage were increased in the DMM + AAV‐shRNA‐NC and DMM + AAV‐circRere + AAV‐sh‐Irf2bpl groups compared with the Sham + AAV‐shRNA‐NC and DMM + AAV‐circRere + AAV‐shRNA‐NC groups (Figure 6C,D), indicating that circRere modulated the progression of DMM‐induced OA in mouse by targeting β‐catenin.

For the rescue experiments, AAV‐sh‐Irf2bpl (commencing 1 week after DMM surgery) and ICG‐001 (commencing 1 week after DMM surgery, once weekly for 7 weeks) were co‐IA injected into the affected knees. Infected efficiency was determined by fluorescence examination of knee sections and qRT‐PCR of cartilage (Figure S6C,D). Significant reductions in the examined manifestations of DMM‐induced OA were observed in DMM + AAV‐sh‐Irf2bpl + ICG‐001 group compared with DMM + AAV‐sh‐Irf2bpl + vehicle group, including cartilage erosion, synovitis, SBP thickness and pain, but not osteophyte maturation (Figure 6E,G and Figure S6E), indicating that IA injection of ICG‐001 for 7 weeks significantly abolished the detrimental effects of AAV‐sh‐Irf2bpl on the progression of DMM‐induced OA, which was consistent with a previous report to a certain extent.^47^ Expression of MMP13, COL2A1 and β‐catenin and TUNEL assays in mouse cartilage of four groups also corroborated above results (Figure 6F,G). Taken together, these results suggest that circRere is involved in the pathogenesis of DMM‐induced OA in mice by targeting miR‐195a‐5p/Irf2bpl/β‐catenin, and the schematic for the article is shown (Figure 6H).

## 4 DISCUSSION

In this study, circRERE was significantly downregulated in OA cartilage. Gain‐and loss‐of‐function experiments in vitro indicated that circRERE affected apoptosis, proliferation, and anabolic and catabolic biomarker synthesis in HCs. Furthermore, circRere overexpression in mouse knee joints reduced the severity of DMM‐induced OA by targeting the axis of miR‐195a‐5p/Irf2bpl/β‐catenin.

Importantly, we showed that the percentage of m6A‐modified circRERE was increased in OA chondrocytes, and the increased m6A level on circRERE may enhance its endoribonucleolytic cleavage by YTHDF2‐HRSP12‐RNase P/MRP. Considering that YTHDF2‐bound m6A‐modified circRNAs with HRSP‐12 binding sites (GGUUC) are preferentially degraded by RNase P/MRP, MOs‐circRERE targeting GGUUC motifs in circRERE were transfected into HCs followed by IL‐1β treatment. Decreased circRERE expression in HCs stimulated with IL‐1β for 6 h was partly reversed by MOs‐circRERE transfection, but not at 12 or 36 h, which may corroborate the preferential degradation of circRNAs in an HRSP12‐dependent manner. Functionally, the increased apoptosis of HCs upon 6 h IL‐1β stimulation was also partially rescued by MOs‐circRERE treatment.

Although the origin of increased m6A‐methylated circRERE in OA chondrocytes is unclear, it is most likely to be connected with METLL3 activity. To validate the effects of METTL3 on m6A modification of circRERE, METTL3 was silenced by siRNA in human OA chondrocytes. Unsurprisingly, METTL3 downregulation significantly decreased the m6A level on circRERE, but had no impact on the expression level of circRERE (Figure S3A‐C), indicating that METTL3 is a crucial m6A methylase for circRERE in HCs. And METTL3 activity in human chondrocytes, may at least in a significant part, be responsible for the status of m6A level on circRERE. These results suggested that METTL3 activity may be critical for cartilage homeostasis. Intriguingly, a recent report by Chen et al showed that the protein and mRNA levels of METTL3 were profoundly increased in fibroblast‐like synoviocytes (FLSs) isolated from the synovium of patients with OA compared with patients without OA, and synovium‐targeted inhibition of METTL3 alleviated the progression of OA in a DMM mouse model.^28^ Thus, further trials studying the roles of m6A in the occurrence and development of OA are warranted. Notably, in our study, the reason that we use MOs‐circRERE to block two GGUUC motifs in circRERE, rather than m6A methylation sites, is though circRERE is m6A‐methylated, there are four possible m6A modification sites in circRERE. And blocking m6A sites of circRERE may influence its function of miRNA sponge.

In order to find associated pathways, we also performed KEGG analysis of differentially expressed mRNAs between the OA and control human cartilage (Figure S5I,J, the same cartilage samples used for circRNA microarray), but we did not find pathways of interest. Growing evidence highlights the need for balanced Wnt/β‐catenin signalling to maintain cartilage homeostasis.^9^, ^32^ Here, we showed that IRF2BPL could drive ubiquitination and degradation of β‐catenin in HCs, thus participating in the regulation of Wnt/β‐catenin signalling in HCs. In addition, WB analysis indicated that knockdown of GSK3β did not abrogate the suppressive effect of IRF2BPL on β‐catenin protein level in human chondrocytes (Figure S5R,S). Thus, IRF2BPL might degrade β‐catenin independent of GSK3β activity. IRF2BPL belongs to the IRF2BP family, which also includes IRF2BP1, IRF2BP2. And IRF2BP2 is reported to be involved in cell homeostasis regulation and promote M2 macrophage polarization,^29^ and it has been reported that synovial macrophage M2 polarization slows down the progression of OA.^48^ The Wnt/β‐catenin signalling is involved in various pathophysiological processes of OA, and it is interesting to note that both excessive activation and inactivation of this pathway seem to contribute to the pathogenesis of OA.^49^ Thus, IRF2BPL might play a role in maintaining a fine balance of Wnt/β‐catenin signalling by driving ubiquitination and degradation of β‐catenin in HCs. Furthermore, in vitro and in vivo experiments in this study indicated the chondroprotective effects of IRF2BPL for the first time.

RIP assays showed that AGO2 was strongly bound to circRERE. Subsequent experiments in HCs showed that circRERE acted as an ceRNA to regulate target mRNA IRF2BPL and subsequent downstream target β‐catenin via sponging miR‐195‐5p. Mouse circRere expresses a homology of human circRERE (94% similarity), and the binding sequences for miR‐195‐5p in two circRNAs are conserved (Figure S4D). Furthermore, the binding of circRere to miR‐195a‐5p was also confirmed by luciferase reporter assays (Figure S4G,H). In addition, the binding sequence between miR‐195‐5p and its target IRF2BPL also shares high conservation across various vertebrates (Figure S5M). Thus, we performed DMM surgery and IA‐injection of AAVs and antagonist in mice to validate the axis of circRere/miR‐195a‐5p/Irf2bpl/β‐catenin.

Given that mass spectrometry analysis following RNA pull‐down in HCs to screen circRERE‐interacting proteins was not conducted, other proteins may interact with circRERE. Due to the existence of predicted ORFs and m6A modification in circRERE, the possibility of circRERE to encode proteins could not be excluded. Therefore, further exploration is needed to discover other possible mechanisms by which circRERE may affect the pathogenesis of OA. Another limitation of this work is that only surgically induced OA model (DMM) was examined, we did not examine other subsets of OA, such as natural age‐related OA. Follow‐up work is needed to test the function of circRERE in other subsets of OA. Furthermore, this study may be improved by employing transgenic mice with a chondrocyte‐specific promoter for circRere, rather than using AAVs to regulate circRere expression.

In conclusion, circRERE downregulation in OA chondrocytes was likely attributed to its increased m6A modification prone to endoribonucleolytic cleavage by YTHDF2‐HRSP12‐RNase P/MRP, and circRERE downregulation led to aberrant β‐catenin ubiquitination and degradation by targeting miR‐195‐5p/IRF2BPL during OA pathogenesis. Thus, concomitant targeting of m6A modification of circRERE and its downstream target miR‐195‐5p/IRF2BPL/β‐catenin might provide a synergistic effect in OA clinical treatment.

## AUTHOR CONTRIBUTIONS

JL conceived this study and supervised the project. YXL conducted most of the experiments and acquired data with the help from YHY, YCL, BW, XYH, LX and WTZ. YXL and YHY contributed to data interpretation and analysis. JL wrote the manuscript. JL, YXL and YHY reviewed the manuscript. All authors approved the final manuscript.

## CONFLICT OF INTEREST

The authors declare no competing interests.

## Supporting information


**APPENDIX S1** Supporting Information

**FIGURE S1** (A) For 13 circRNAs, three primers of each were designed to amplify them in cDNA and gDNA of human chondrocytes by RT‐PCR and agarose gel electrophoresis analysis. (B) Relative expressions of circZFHX4, circTBCK, circTENM3, circRERE, circARHGAP5 and RERE in human control and OA‐Damaged cartilage were detected by qRT‐PCR and agarose gel electrophoresis analysis (n=20). *p<0.05, **p<0.01 by Mann‐Whitney U test. (C) Pairwise alignment of the human circRERE and mouse circRere sequences. (D) Relative expressions of circRere and Rere in total RNA of MCs treated with or without RNase R (n=3). ***p<0.001 by two‐tailed unpaired t test. (E) Relative expressions of circRere and Rere in MCs treated with Actinomycin D (n=3). ***p<0.001 by two‐way ANOVA with Tukey's post hoc test. (F) FISH of circRere in MCs. Scale bar, 20µm. Data are presented as mean ± SEM.
**Figure S2** (A) β‐galactosidase staining and quantitative analysis for the β‐gal‐positive HCs infected with Ad‐circRERE or Ad‐vector and treated with doxorubicin (Doxo, 100 nM, 5 days). n=3. Scale bar, 200µm. NS, no significance. ***p<0.001 by One‐way ANOVA with Tukey's post hoc test. (B) Overview of DMM surgery, IA‐injection with AAV‐vector or AAV‐circRere, pain assays and sampling. (C) Relative circRere expression in mouse cartilage from Sham+AAV‐vector, DMM+AAV‐vector and DMM+AAV‐circRere groups. n=4. **p<0.01 by Brown‐Forsythe and Welch ANOVA test followed by Dunnett's T3 multiple comparison test. Data are presented as mean ± SEM.
**Figure S3** (A) Efficiency of METTL3 downregulation in human OA chondrocytes (isolated from damaged cartilage of OA patients) by si‐METTL3 (n=3). **p<0.01 by two‐tailed unpaired t test. (B) The percentage of m6A‐modified circRERE upon METTL3 downregulation in human OA chondrocytes (isolated from damaged cartilage of OA patients) (n=3). ***p<0.05 by two‐way ANOVA with Tukey's post hoc test. (C) Relative expression of circRERE upon METTL3 downregulation in human OA chondrocytes (isolated from damaged cartilage of OA patients) (n=3). NS by two‐tailed unpaired t test. Data are presented as mean ± SEM.
**Figure S4** (A) Number of RBP sites between RBPs and circRERE. (B) FMRP, IGF2BP3, EIF4A3, and DGCR8 RIP assays and subsequent qRT‐PCR analysis were performed to detect CO‐RIPed‐circRERE expression in HCs infected with Ad‐vector or Ad‐circRERE (n=6). *p<0.05, *p<0.01 by two‐tailed unpaired t test or Welch's t test. (C) Schematic to show the overlapping of the target miRNAs of circRERE, as predicted by miRanda, TargetScan and Arraystar's proprietary program. (D) The potentially conserved binding sites between two circRNAs (hsa_circRERE, mmu_circRere) and miR‐195‐5p, predicted by miRanda and TargetScan. The potential complementary residues are shown in blue. (E) Pairwise sequence alignment of hsa_circRERE and mmu_circRere, and the sequences highlighted in red are targeted by miR‐195‐5p. (F) Schematic of luciferase reporter vectors containing wild‐type (WT) or mutant (Mut) putative miR‐195‐5p binding sites of circRERE. (G) Schematic of luciferase reporter vectors containing wild‐type (WT) or mutant (Mut) putative miR‐195a‐5p binding sites of mmu_circRere. (H) Relative luciferase activity of WT or MUT circRere luciferase reporter vector co‐transfected with miR‐195‐5p mimics or negative control (n=4). ***p<0.001by two‐tailed unpaired t test. (I) Quantification of Figure 4H (n=3). *p<0.05 by two‐way ANOVA with Tukey's post hoc test. (J, K) HCs were transfected with miR‐195‐5p inhibitor and stimulated with IL‐1β (36h). Then proliferation was determined by EdU assays (n=3). One‐way ANOVA with Tukey's post hoc test. (L) Quantification of Figure 4L (n=3). *p<0.05 by two‐way ANOVA with Tukey's post hoc test. (M) Relative expressions of circRere and miR‐195a‐5p in knee cartilage from Sham+AAV‐miR‐NC, DMM+AAV‐miR‐NC, DMM+AAV‐circRere+AAV‐miR‐NC and DMM+AAV‐circRere+AAV‐miR‐195a‐5p groups (n=4). **p<0.01 by Brown‐Forsythe and Welch ANOVA test followed by Dunnett's T3 multiple comparison test. NS, no significance. Data are presented as mean ± SEM.
**Figure S5** (A) Volcano plot illustrating the statistical significance of differentially expressed mRNAs between human OA and control cartilage. (B) Scatter plot of differentially expressed mRNAs. (C‐E) Gene Ontology (GO) analysis of upregulated genes in OA cartilage. (F‐H) GO analysis of downregulated genes in OA cartilage. (I) Kyoto Encyclopedia of Genes and Genomes (KEGG) analysis of upregulated genes in OA cartilage. (J) KEGG analysis of downregulated genes in OA cartilage. (K) Schematic to show the overlapping of the target mRNAs of miR‐195‐5p, as predicted by PITA, miRanda, Pictar, TargetScan and downregulated mRNAs in OA cartilage identified by mRNA microarray. (L) QRT‐PCR for relative expression of predicted target mRNAs of miRNA‐195‐5p in HCs infected with Ad‐sh‐con or Ad‐sh‐circRERE. n=6. ***p<0.001 by two‐tailed unpaired t test. (M) Putative miR‐195‐5p.
**Figure S6** (A) Relative expressions of circRere and Irf2bpl in knee cartilage from Sham+AAV‐shRNA‐NC, DMM+AAV‐shRNA‐NC, DMM+AAV‐circRere+AAV‐shRNA‐NC and DMM+AAV‐circRere+AAV‐sh‐Irf2bpl groups (n=4). **p<0.01, ***p<0.001 by one‐way ANOVA with Tukey's post hoc test. (B) Scoring of osteophyte maturity in four groups. one‐way ANOVA with Tukey's post hoc test. (C) To investigate the infected efficiency of AAVs, representative knee cartilage fluorescence (GFP) images in knee sections from DMM+AAV‐sh‐Irf2bpl+vehicle and DMM+AAV‐sh‐Irf2bpl+ICG‐001 groups were obtained by a confocal microscope. Scale bar, 200µm. (D) Relative expression of Irf2bpl in knee cartilage from Sham+vehicle, DMM+vehicle, DMM+AAV‐sh‐Irf2bpl+vehicle and DMM+AAV‐sh‐Irf2bpl+ICG‐001 groups (n=4). *p<0.05 by one‐way ANOVA with Tukey's post hoc test. (E) Scoring of osteophyte maturity in four groups (n=6). *p<0.05 by one‐way ANOVA with Tukey's post hoc test. NS, no significance. Data are presented as mean ± SEM.

## Data Availability

The data that support the findings of this study are available from the corresponding author upon reasonable request.
