# Supplementary material for: N6 ‐methyladenosine‐modified circRNA RERE modulates osteoarthritis by regulating β‐catenin ubiquitination and degradation
Source: Cell Prolif. 2022 Jun 22;56(1):e13297. doi: 10.1111/cpr.13297 (PMC9816929; doi:10.1111/cpr.13297)
Supplement: Supplementary file 1 — APPENDIX S1 Supporting Information [file CPR-56-e13297-s001.docx]

**EXPERIMENTAL PROCEDURES**

**N^6^-methyladenosine-modified circRNA RERE modulates osteoarthritis by regulating β-Catenin ubiquitination and degradation**

Yuxi Liu^1^, Yunhan Yang^2^, Yucheng Lin^1^, Bing Wei^1^, Xinyue Hu^1^, Li Xu^1^, Weituo Zhang^1^, Jun Lu^1^

**Human subjects**

Human cartilage samples were collected according to protocols approved by the Ethics Committee of affiliated Zhongda Hospital of Southeast University (2020ZDSYLL222-P01). Written informed consent was obtained from all subjects. A total of 100 human OA cartilage samples were obtained from OA patients undergoing knee arthroplasty. A total of 40 normal control cartilage samples were obtained from amputees without history of arthritic diseases or patients suffered from femoral neck fractures or comminuted proximal humeral fractures with intact femoral or humeral heads as previously described^1,2^.

**Microarray analysis**

Sample labeling and array hybridization of microarray were performed according to the manufacturer’s protocol. Briefly, for the circRNA microarray, total RNAs were digested with RNase R (Epicentre) to remove linear RNAs and enrich circular RNAs. Then, the enriched circular RNAs were amplified and transcribed into fluorescent cRNA utilizing a random priming method (Arraystar Super RNA Labeling Kit; Arraystar). The labeled cRNAs were hybridized onto the Arraystar Human circRNA Array V2 (8 × 15 K, Arraystar). After washing, the arrays were scanned by the Agilent Scanner G2505C. Agilent Feature Extraction software (version 11.0.1.1) was used to analyze acquired array images. The basic steps of mRNA microarray are similar to that of circRNA microarray without RNase R digestion of total RNA. All of the microarray analysis was performed by Aksomics Inc. (Shanghai, China). The Gene Expression Omnibus (GEO) accession code for the circRNA microarray in this manuscript is GSE178724, for mRNA microarray is GSE178557.

**Bioinformatics analysis**

The miRNA targets of circRERE were predicted by TargetScan and miRanda^3-5^. The potential target genes of miR-195-5p were predicted using mRNA microarray results, TargetScan, miRanda, PITA and PicTar^3-6^. The potential Ago2 and other RBP binding sites of circRERE were obtained using Circular RNA Interactome^7^.

**RNA extraction and quantitative Real-time PCR**

Total RNA was extracted from the cartilage tissue samples or cultured chondrocytes with TRIzol reagent (Invitrogen, Carlsbad, CA, USA) according to the manufacturer’s protocol. After being tested by a SmartSpec Plus spectrophotometer (Bio-Rad), RNA was subjected to reverse transcription using Hiscript RT SuperMix Kit (Vazyme, Nanjing, China) for mRNAs or miRNA 1st Strand cDNA Synthesis Kit (Vazyme, Nanjing, China) for miRNAs. Real-time PCR was performed using AceQ qPCR SYBR Green Master Mix (Vazyme, Nanjing, China) and detected by StepOnePlusTM Real-Time PCR System (Applied Biosystems, USA). The primers sequences are shown in Supplementary Table S1.

**Actinomycin D and RNase R treatment**

To assess the stability of circRERE/circRere and its linear isoform, human/mouse chondrocytes were exposed to Actinomycin D (2 μg/ml) at the indicated time point. After removal of actinomycin D, the expression of *RERE/Rere* and circRERE/circRere was analyzed by qRT-PCR. For RNase R treatment, total RNAs were incubated with or without 3 U/μg RNase R (Epicenter Technologies, USA) for 25 min at 37°C, and the resulting RNA was subsequently purified using the RNeasy MinElute Cleanup Kit (Qiagen, Germany).

**RNA interference and overexpression**

For circRNA interference, three different siRNAs were designed and tested for circRERE in HCs (RiboBio, Guangzhou, China). The most efficient siRNA#1 sequence was chosen to make an adenovirus expressing shRNA#1 against human circRERE (Ad-sh-circRERE). The adenoviruses Ad-sh-circRERE, Ad-circRERE, Ad-miR-195-5p, and Ad-IRF2BPL were purchased from Vigene Biosciences (Jinan, China). Chondrocytes were infected with adenoviruses at MOI of 200-400 within 12 h. Specific inhibitor or mimic (RiboBio, Guangzhou, China) was used to inhibit or induce miR-195-5p expression, respectively. Cell transfection with plasmids was achieved using Lipofectamine 2000 or 3000 transfection reagent (ThermoFisher). RiboFECT™ CP (RiboBio, Guangzhou, China) was used for the siRNAs, miR-195-5p inhibitor and mimic transfection. SiRNA sequences are shown in Supplementary Table S1.

**Western blotting**

Total human chondrocyte lysates with various treatments were prepared in RIPA lysis buffer (Beyotime, China), and the protein concentration of the lysates was determined using a BCA protein quantification asssay (KeyGEN BioTECH, China). Extractions were loaded and separated by SDS-PAGE (6% or 10%). Next, the proteins were electrophoretically transferred onto polyvinylidene fluoride membranes. The membranes were blocked for 2 h with 5% bovine serum albumin (BSA) in TBST and incubated with primary antibodies for MMP13 (1:1000, 18165-1-AP, Proteintech), ADAMTS5 (1:1000, A02802-1, Boster), COL2A1 (1:500, BA0533, Boster), Aggrecan (1:1000, ab36861, Abcam), IRF2BPL (1:1000, ab221099, Abcam; or 1:100, sc-514772, Santa Cruz), β-catenin (1:1000, 66379-1-Ig, Proteintech; or #9562, CST), Active β-Catenin (1:1000, #4270, CST), YTHDF2 (1:1000, 24744-1-AP, Proteintech), FTO (1:1000, 27226-1-AP, Proteintech), METTL3 (1:1000, 15073-1-AP, Proteintech), GAPDH (1:2000, 60004-1-Ig, Proteintech), GSK3β (1:1000, 22104-1-AP, Proteintech) and β-actin (1:2000, 66009-1-Ig, Proteintech) overnight at 4 °C. Membranes were then incubated with HRP-conjugated affinipure goat anti-rabbit or mouse IgG(H+L) secondary antibody (1:2,000, SA00001-2 or SA00001-1, Proteintech) for 2h. After washing, signals were detected by an enhanced chemiluminescence system (ECL) detection system. The experiments were performed at least in triplicate.

**Experimental osteoarthritis in mice**

Wild type C57BL/6 mice were purchased from GemPharmatech (Nanjing, China) and animal handling and experimental procedures were approved by the Institutional Animal Care and Use Committee at the Medical School of Southeast University (No.20200921006). A model of osteoarthritis was induced by the destabilization of the medial meniscus (DMM) in the right knee of 10-week-old male mice as previously described^8^. Briefly, after anaesthesia with isoflurane, mouse joint surgery on right knee was performed by transecting the medial menisco-tibial ligament anchoring medial meniscus to tibial plateau. Sham surgery, without the transection of ligament, was performed following the same procedure. Mice were allowed to move in the cage without immobilization after surgery. After corresponding *in vivo* experiments, mice were sacrificed at 5 or 8 weeks post-DMM, and the knee joints and sera were collected and stored at -80°C. All mice were maintained under pathogen-free conditions with free access to food and water. For all experiments, mice were randomly assigned to each experimental group.

**Articular injection in mice**

For the therapeutic experiments, adeno-associated viruses (AAV) overexpressing circRere (AAV-circRere, 10 μl; 4.29×10^13^ vg/ml) was IA-injected into the operated knees in DMM mice (commencing 1 week after DMM surgery) as previously described^9^, and intra-articular (IA) injection of empty AAV (AAV-vector, 10 μl; 6.62×10^13^ vg/ml) was used as control. For the deprivation experiments, a mixture including 5 μl AAV-circRere and 5 μl AAV overexpressing miR-195a-5p (AAV-miR-195a-5p, 3.59×10^13^ vg/ml) or 5 μl AAV-circRere and 5 μl AAV-sh-Irf2bpl (1.54×10^13^ vg/ml) was co-injected (IA) into the affected knees of DMM mice (commencing 1 week after DMM surgery), and a mixture including 5 μl AAV-circRere and 5 μl AAV-miR-NC (negative control, 2.63×10^13^ vg/ml) or 5 μl AAV-circRere and 5 μl AAV-shRNA-NC (negative control, 8.07×10^13^ vg/ml) was used as control. In addition, in order to explore whether circRere modulated OA pathogenesis *in vivo* through β-catenin signalling, DMM mice IA-injected with AAV-sh-Irf2bpl (commencing 1 week after DMM surgery) were co-IA-injected with ICG-001 (25 mg/kg/week, Selleck), an inhibitor of β-catenin, for 7 weeks (once a week) as previously described^10^. AAV-circRere (mouse, GFP-tagged), AAV-miR-195a-5p (mouse, GFP-tagged) and AAV-sh-Irf2bpl (mouse, GFP-tagged) were purchased from Vigene Biosciences (Jinan, China). The infected efficiency of AAV in cartilage and synovium of mice knee was detected by histology (fluorescence detected by a confocal microscope), and qRT-PCR.

**Histology, TUNEL staining, Immunohistochemistry and** **Von Frey assay**

Human cartilage was frozen and sectioned at 10μm. Mouse knee joint specimens were fixed in 4% paraformaldehyde and decalcified using 0.5 M EDTA for 2 weeks. After dehydration, the samples were embedded in paraffin. Continuous frontal sections of paraffin-embedded knee joint tissues were sectioned at 5μm, and histological OA parameters in mice (cartilage deterioration, synovitis score, osteophyte maturity, and subchondral bone plate (SBP) thickness) were determined using Safranin-O/fast green or HE staining. Cartilage deterioration was scored by blinded observers using the Osteoarthritis Research Society International (OARSI) grading system, a validated scoring system (grade 0-6)^11^. Synovitis was scored by a 0–3 grading system based on three features (leucocytic infiltration, cellular density of synovial stroma and enlargement of synovial lining cell layer) as described previously^12^. Osteophyte formation was identified by Safranin-O/fast green staining, and osteophyte maturity (grade 0-3) was scored as previously described^13^. In order to assess subchondral bone sclerosis, we measured the SBP thickness as previously reported^14^. All histological analysis scores are presented as the mean grade of the ten sections for each mouse, and a representative Safranin-O/fast green or HE staining image was presented. TUNEL staining was performed using the riboAPOTM One-Step TUNEL Apoptosis Kit (Ribobio, Guangzhou, China) according to the manufacturer’s protocol. For immunohistochemistry (IHC) of mouse joint sections, the sections were deparaffinized, hydrated, and incubated at 4 °C with primary antibody to MMP13, COL2A1 and β-catenin (1:200) overnight and for 2 h at room temperature with HRP-conjugated secondary antibody. Afterwards, slides were processed with a DAB horseradish peroxidase colour development kit (Beyotime, China) and counterstained with hematoxylin. OA-associated pain in the different groups was measured by von Frey filaments.

**SA-****β-galactosidase staining**

SA-β-gal staining was performed using a Cell Senescence β-Galactosidase Staining Kit (40754ES60, Yeasen, Shanghai, China) according to the manufacturer's instructions. Briefly, cells were incubated with the kit’s staining mixture for 16 h at 37 °C. Senescent cells (SnCs) were identified as blue-stained cells under light microscopy.

**Chondrocyte isolation and culture**

Human control chondrocytes were isolated from cartilage of amputees. Human OA chondrocytes were isolated from damaged-OA cartilage of OA patients undergoing knee arthroplasty. Human chondrocytes abbreviated as HCs in the article were isolated from intact-OA cartilage of OA patients undergoing knee arthroplasty as previously described^15^. The primary mouse chondrocytes were collected from the femoral condyles and tibial plateaus of 5 days old C57BL/6 mice by sequential enzymatic digestions as described by Gosset et al^16^. Briefly, the cartilage tissue was cut into 1-mm^3^ pieces, washed 3 times with PBS, and digested on a shaker for 16 hours with 0.2% collagenase type II (Sigma-Aldrich, St. Louis, MO, USA) in Dulbecco’s modified Eagle’s medium (DMEM, Gibco; 1g/L glucose for mouse primary chondrocytes; 4.5g/L glucose for human chondrocytes) with antibiotics at 37 °C under 5% CO^22,17^. After digestion, the filtrate was passed through a 100-μm cell strainer, then cells were rinsed 3 times with sterile PBS, resuspended and cultured in DMEM with 10% FBS at 37 °C in a humidified atmosphere of 5% CO^2^ and 95% air. Passage 0 cells were used for analyses.

**Immunofluorescence**

Immunofluorescence staining was performed in chondrocytes or cartilage from human or mouse. Briefly, cells or sections were permeabilized with 0.1% Triton X-100, blocked with 5% BSA and incubated with MMP13, Aggrecan, ADAMTS5, COL2A1, IRF2BPL or m6A (ab151230, Abcam; or 202003, Synaptic Systems) antibodies (1:200) at 4 °C overnight. After being washed with PBS, they were stained with Alexa Fluor 594 AffiniPure Goat Anti-Rabbit IgG (H+L), or Alexa Fluor 594 AffiniPure Goat Anti-Mouse IgG (H+L), or Alexa Fluor 647 AffiniPure Goat Anti-Rabbit IgG (H+L) (1:200, 33112ES60, 33212ES60, 33113ES60, Yeasen, Shanghai, China). DAPI was used for nuclear staining. Immunofluorescence images were obtained by a confocal microscope (Olympus FV3000).

**RNA fluorescence *in situ* hybridization (FISH****)**

The FISH assay was performed in chondrocytes or cartilage from humans or mice. Cy3-labeled circRERE probes, Cy3-labeled circRere probes and FAM-labeled miR-195-5p probes were designed and synthesized by RiboBio (Guangzhou, China). The hybridization was performed by a Fluorescence in situ Hybridization Kit according to the manufacturer’s instructions. For FISH-IF staining, the samples were prehybridized in pre-hybridization buffer for 30 min before overnight incubation at 37°C in hybridization buffer containing cy3-labeled circRERE probes (RiboBio, Guangzhou, China). After completion of *in situ* hybridization, the samples were blocked for 1 hour at 21-23°C in 1% BSA/0.1% Triton X-100 in PBS (wt/v for BSA and v/v for Triton X-100). Then samples were incubated with the primary antibody anti-m6A (1:200, ab151230, Abcam; or 202003, Synaptic Systems), or anti-IRF2BPL (1:200, ab221099, Abcam) overnight at 4°C; washed three times with 1×PBS; and incubated with the secondary antibody Alexa Fluor 647 AffiniPure Goat Anti-Rabbit IgG (H+L) (1:200, 33113ES60, Yeasen, Shanghai, China) for 1 hour. DAPI was used for nuclear staining. Images were detected and analyzed using an FV31S-SW Viewer on a confocal laser scanning microscope (Olympus, FV3000).

**Flow cytometry assay**

The human chondrocytes (HCs) used for flow cytometry analysis were isolated from intact-OA cartilage of OA patients undergoing knee arthroplasty. And passage 0 cells of human chondrocytes were used for flow experiments. Chondrocytes receiving different treatments were collected using trypsin (25300120, Gibco) and washed with PBS. Apoptosis was detected using an Annexin V-FITC/PI or Annexin V-APC/PI Apoptosis Kit (Multi Sciences, Hangzhou, China). The collected cells were stained with annexin V-FITC or Annexin V-APC and propidium iodide for 15 min according to the manufacturer's instructions. Apoptosis was detected with an Attune NxT flow cytometer (ThermoFisher).

**RNA immunoprecipitation assay**

RNA immunoprecipitation (RIP) assay was performed using a Magna RIP™ RNA-binding protein immunoprecipitation kit (Millipore, Bedford, MA, USA) according to the manufacturer’s guidelines. Briefly, after being washed, the magnetic beads were incubated with 5 μg antibody anti-AGO2 (ab32381, Abcam), anti-m6A (ab151230, Abcam; or 202003, Synaptic Systems), anti-FMRP (ab264380, Abcam), IGF2BP3 (#57145, CST; or ab177477, Abcam), EIF4A3 (ab32485, Abcam) or DGCR8 (ab191875, Abcam) in RIP washing buffer and incubated for 30 minutes at room temperature to generate antibody-coated beads. Human chondrocytes were lysed in 100 μl RIP lysis buffer and then diluted with 900 μl RIP immunoprecipitation buffer and incubated with the antibody-coated beads overnight at 4 °C. After that, beads were washed six times using RIP wash buffer. The immunoprecipitates were treated with Proteinase K at 55 °C for 30 minutes. And the co-precipitated RNA was extracted using TRIzol regent (Invitrogen), followed by qRT-PCR. IgG (regarded as a negative control) and Input (regarded as a positive control) were assayed simultaneously.

**Morpholino oligos (MOs) treatment**

For treatment with MOs in HCs, two MOs targeting GGUUC motifs in circRERE (MOs-circRERE, GeneTools, USA) were added to culture media at a final concentration of 10 μM along with Endoporter (GeneTools, USA) and incubated for 24 hours. The sequence of two MOs-circRERE is as follows: GGAACCTCAGATTGACGGTAGTACC; TGTCGATCCTGAACCAAATGCTGAT.

**RNA Pull-down assay with biotinylated circRERE/circRere probe**

The RNA pull-down assay was performed with biotinylated probe as previously described^18-20^. Briefly, for circRERE pulled down miRNAs, the biotinylated-circRERE/circRere probe was incubated with C-1 magnetic beads (65001, Invitrogen) to generate probe-coated beads. The human or mouse chondrocytes were lysed in co-IP buffer, then lysates were incubated with the probe-coated beads at 4 °C overnight. The beads were washed 5 times briefly with co-IP buffer, and the RNA complexes bound to the beads were eluted and extracted for qRT-PCR analysis. For miR-195-5p pulled down circRERE, human chondrocytes with circRERE overexpression were transfected with biotinylated miR-195-5p mimics or mutant. The cells were harvested 48 h after transfection, lysed, sonicated, and incubated with C-1 magnetic beads. Subsequently, the biotin-coupled RNA complex was washed, and RNA was extracted for qRT-PCR. The sequence of biotinylated human circRERE probe was 5'-GTTGTGGACCTTGTCTCAGG-3'. The sequence of biotinylated mouse circRere probe was 5'-GCTGTGGACCTTGTCTCAGG-3'. The scramble sequence was 5'-TATGCCGGATCGTGGTTCTG-3'. The sequence of biotinylated miR-195-5p wild type (WT) was 5'-UAGCAGCACAGAAAUAUUGGC-3'. The sequence of biotinylated miR-195-5p mutant (Mut) was 5'-GGCAGCAUGAUAACAUUGACA-3'.

**Luciferase activity assays**

For circRERE (or circRere) and miR-195-5p, either wild-type or mutant CircRERE (or circRere) fragments were inserted into firefly_Luciferase vector (Genechem, Shanghai, China). For IRF2BPL and miR-195-5p, either wild-type or mutant IRF2BPL 3’UTR fragments were inserted into firefly_Luciferase vector (Genechem, Shanghai, China). Briefly, circRERE-WT or circRERE-Mut plasmids were co-transfected with miR-195-5p mimic or control (Con) into cells. CircRere-WT or circRere-Mut plasmids were co-transfected with miR-195a-5p mimic or control (Con) into cells. IRF2BPL 3’UTR-WT or IRF2BPL 3’UTR-Mut plasmids were co-transfected with miR-195-5p mimic or control (Con) into cells. After incubation, the luciferase activity was analyzed with Dual Luciferase Reporter Assay Kit (Vazyme, Nanjing, China).

**Statistical analysis**

Statistical analysis was performed with GraphPad Prism 8 or SPSS Statistics 24. Data distribution was tested with Shapiro-Wilk test. The homogeneity of variance was tested by Brown-Forsythe test. Statistical analysis between two groups was performed by two-tailed unpaired Student’s t test (normal distribution and equal variances), Welch’s t test (unequal variances), or Mann-Whitney U test (non-normal distribution) or two-tailed Wilcoxon matched-pairs signed rank test. One-way or two-way ANOVA analysis followed by the Tukey’s post-hoc test or Brown-Forsythe and Welch ANOVA test followed by Dunnett’s T3 multiple comparison test was used for multiple group comparisons. For all analysis, results were considered statistically different if p was <0.05 between groups.

**REFERENCES**

1. Coutinho de Almeida R, Ramos YFM, Mahfouz A, et al. RNA sequencing data integration reveals an miRNA interactome of osteoarthritis cartilage. *Ann Rheum Dis.* 2019;78(2):270-277.

2. Zhu X, Chen F, Lu K, Wei A, Jiang Q, Cao W. PPARgamma preservation via promoter demethylation alleviates osteoarthritis in mice. *Ann Rheum Dis.* 2019;78(10):1420-1429.

3. Agarwal V, Bell GW, Nam JW, Bartel DP. Predicting effective microRNA target sites in mammalian mRNAs. *Elife.* 2015;4.

4. Betel D, Wilson M, Gabow A, Marks DS, Sander C. The microRNA.org resource: targets and expression. *Nucleic Acids Res.* 2008;36(Database issue):D149-153.

5. Kertesz M, Iovino N, Unnerstall U, Gaul U, Segal E. The role of site accessibility in microRNA target recognition. *Nat Genet.* 2007;39(10):1278-1284.

6. Krek A, Grun D, Poy MN, et al. Combinatorial microRNA target predictions. *Nat Genet.* 2005;37(5):495-500.

7. Dudekula DB, Panda AC, Grammatikakis I, De S, Abdelmohsen K, Gorospe M. CircInteractome: A web tool for exploring circular RNAs and their interacting proteins and microRNAs. *RNA Biol.* 2016;13(1):34-42.

8. Glasson SS, Blanchet TJ, Morris EA. The surgical destabilization of the medial meniscus (DMM) model of osteoarthritis in the 129/SvEv mouse. *Osteoarthritis Cartilage.* 2007;15(9):1061-1069.

9. Yang Y, Shen P, Yao T, et al. Novel role of circRSU1 in the progression of osteoarthritis by adjusting oxidative stress. *Theranostics.* 2021;11(4):1877-1900.

10. Li K, Zhang Y, Zhang Y, et al. Tyrosine kinase Fyn promotes osteoarthritis by activating the beta-catenin pathway. *Ann Rheum Dis.* 2018;77(6):935-943.

11. Glasson SS, Chambers MG, Van Den Berg WB, Little CB. The OARSI histopathology initiative - recommendations for histological assessments of osteoarthritis in the mouse. *Osteoarthritis Cartilage.* 2010;18 Suppl 3:S17-23.

12. Krenn V, Morawietz L, Burmester GR, et al. Synovitis score: discrimination between chronic low-grade and high-grade synovitis. *Histopathology.* 2006;49(4):358-364.

13. Little CB, Barai A, Burkhardt D, et al. Matrix metalloproteinase 13-deficient mice are resistant to osteoarthritic cartilage erosion but not chondrocyte hypertrophy or osteophyte development. *Arthritis Rheum.* 2009;60(12):3723-3733.

14. Das Neves Borges P, Vincent TL, Marenzana M. Automated assessment of bone changes in cross-sectional micro-CT studies of murine experimental osteoarthritis. *PLoS One.* 2017;12(3):e0174294.

15. Choi WS, Yang JI, Kim W, et al. Critical role for arginase II in osteoarthritis pathogenesis. *Ann Rheum Dis.* 2019;78(3):421-428.

16. Gosset M, Berenbaum F, Thirion S, Jacques C. Primary culture and phenotyping of murine chondrocytes. *Nat Protoc.* 2008;3(8):1253-1260.

17. Gagarina V, Gabay O, Dvir-Ginzberg M, et al. SirT1 enhances survival of human osteoarthritic chondrocytes by repressing protein tyrosine phosphatase 1B and activating the insulin-like growth factor receptor pathway. *Arthritis Rheum.* 2010;62(5):1383-1392.

18. Yu J, Xu QG, Wang ZG, et al. Circular RNA cSMARCA5 inhibits growth and metastasis in hepatocellular carcinoma. *J Hepatol.* 2018;68(6):1214-1227.

19. Zheng Q, Bao C, Guo W, et al. Circular RNA profiling reveals an abundant circHIPK3 that regulates cell growth by sponging multiple miRNAs. *Nat Commun.* 2016;7:11215.

20. Lal A, Thomas MP, Altschuler G, et al. Capture of microRNA-bound mRNAs identifies the tumor suppressor miR-34a as a regulator of growth factor signaling. *PLoS Genet.* 2011;7(11):e1002363.
