# Supplementary material for: N6 ‐methyladenosine‐modified circRNA RERE modulates osteoarthritis by regulating β‐catenin ubiquitination and degradation
Source: Cell Prolif. 2022 Jun 22;56(1):e13297. doi: 10.1111/cpr.13297 (PMC9816929; doi:10.1111/cpr.13297)
Supplement: Supplementary file 2 — FIGURE S1 (A) For 13 circRNAs, three primers of each were designed to amplify them in cDNA and gDNA of human chondrocytes by RT‐PCR and agarose gel electrophoresis analysis. (B) Relative expressions of circZFHX4, circTBCK, circTENM3, circRERE, circARHGAP5 and RERE in human control and OA‐Damaged cartilage were detected by qRT‐PCR and agarose gel electrophoresis analysis (n=20). *p<0.05, **p<0.01 by Mann‐Whitney U test. (C) Pairwise alignment of the human circRERE and mouse circRere sequences. (D) Relative expressions of circRere and Rere in total RNA of MCs treated with or without RNase R (n=3). ***p<0.001 by two‐tailed unpaired t test. (E) Relative expressions of circRere and Rere in MCs treated with Actinomycin D (n=3). ***p<0.001 by two‐way ANOVA with Tukey's post hoc test. (F) FISH of circRere in MCs. Scale bar, 20µm. Data are presented as mean ± SEM. Figure S2 (A) β‐galactosidase staining and quantitative analysis for the β‐gal‐positive HCs infected with Ad‐circRERE or Ad‐vector and treated with doxorubicin (Doxo, 100 nM, 5 days). n=3. Scale bar, 200µm. NS, no significance. ***p<0.001 by One‐way ANOVA with Tukey's post hoc test. (B) Overview of DMM surgery, IA‐injection with AAV‐vector or AAV‐circRere, pain assays and sampling. (C) Relative circRere expression in mouse cartilage from Sham+AAV‐vector, DMM+AAV‐vector and DMM+AAV‐circRere groups. n=4. **p<0.01 by Brown‐Forsythe and Welch ANOVA test followed by Dunnett's T3 multiple comparison test. Data are presented as mean ± SEM. Figure S3 (A) Efficiency of METTL3 downregulation in human OA chondrocytes (isolated from damaged cartilage of OA patients) by si‐METTL3 (n=3). **p<0.01 by two‐tailed unpaired t test. (B) The percentage of m6A‐modified circRERE upon METTL3 downregulation in human OA chondrocytes (isolated from damaged cartilage of OA patients) (n=3). ***p<0.05 by two‐way ANOVA with Tukey's post hoc test. (C) Relative expression of circRERE upon METTL3 downregulation in human OA chondrocytes (isolated fr [file CPR-56-e13297-s002.docx]

**Supplementary Material**

**N^6^-methyladenosine-modified circRNA RERE modulates osteoarthritis by regulating β-Catenin ubiquitination and degradation**

Yuxi Liu^1^, Yunhan Yang^2^, Yucheng Lin^1^, Bing Wei^1^, Xinyue Hu^1^, Li Xu^1^, Weituo Zhang^1^, Jun Lu^1^

**Supplementary Figures and Table**

**Supplementary Figures and Legends**

**Figure S1**

**
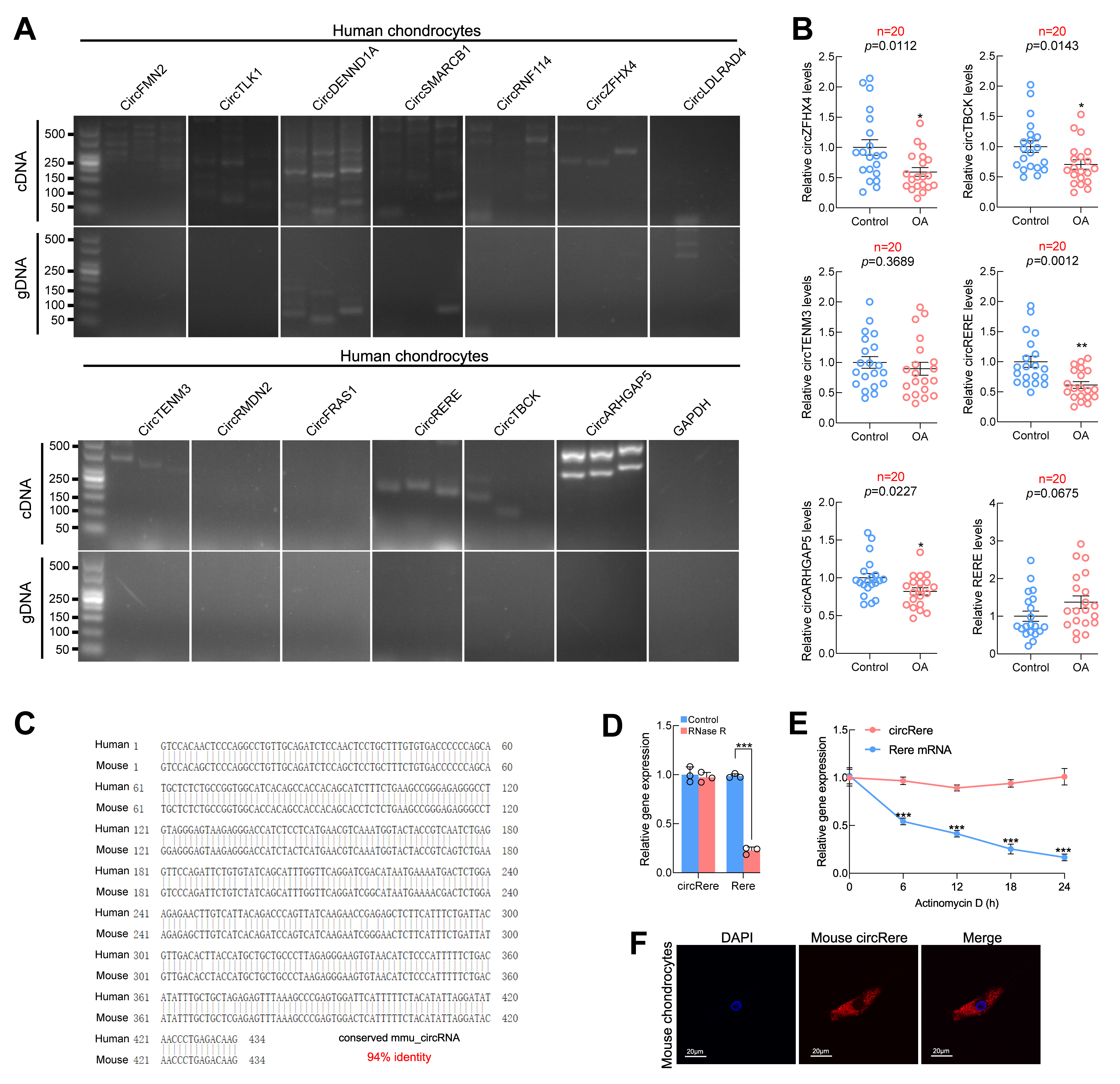
**

**FIGURE S1** (A) For 13 circRNAs, three primers of each were designed to amplify them in cDNA and gDNA of human chondrocytes by RT-PCR and agarose gel electrophoresis analysis. (B) Relative expressions of circZFHX4, circTBCK, circTENM3, circRERE, circARHGAP5 and *RERE* in human control and OA-Damaged cartilage were detected by qRT-PCR and agarose gel electrophoresis analysis (n=20). *p<0.05, **p<0.01 by Mann-Whitney U test. (C) Pairwise alignment of the human circRERE and mouse circRere sequences. (D) Relative expressions of circRere and *Rere* in total RNA of MCs treated with or without RNase R (n=3). ****p*<0.001 by two-tailed unpaired t test. (E) Relative expressions of circRere and *Rere* in MCs treated with Actinomycin D (n=3). ****p*<0.001 by two-way ANOVA with Tukey’s post hoc test. (F) FISH of circRere in MCs. Scale bar, 20µm. Data are presented as mean ± SEM.

**Figure S2**

**
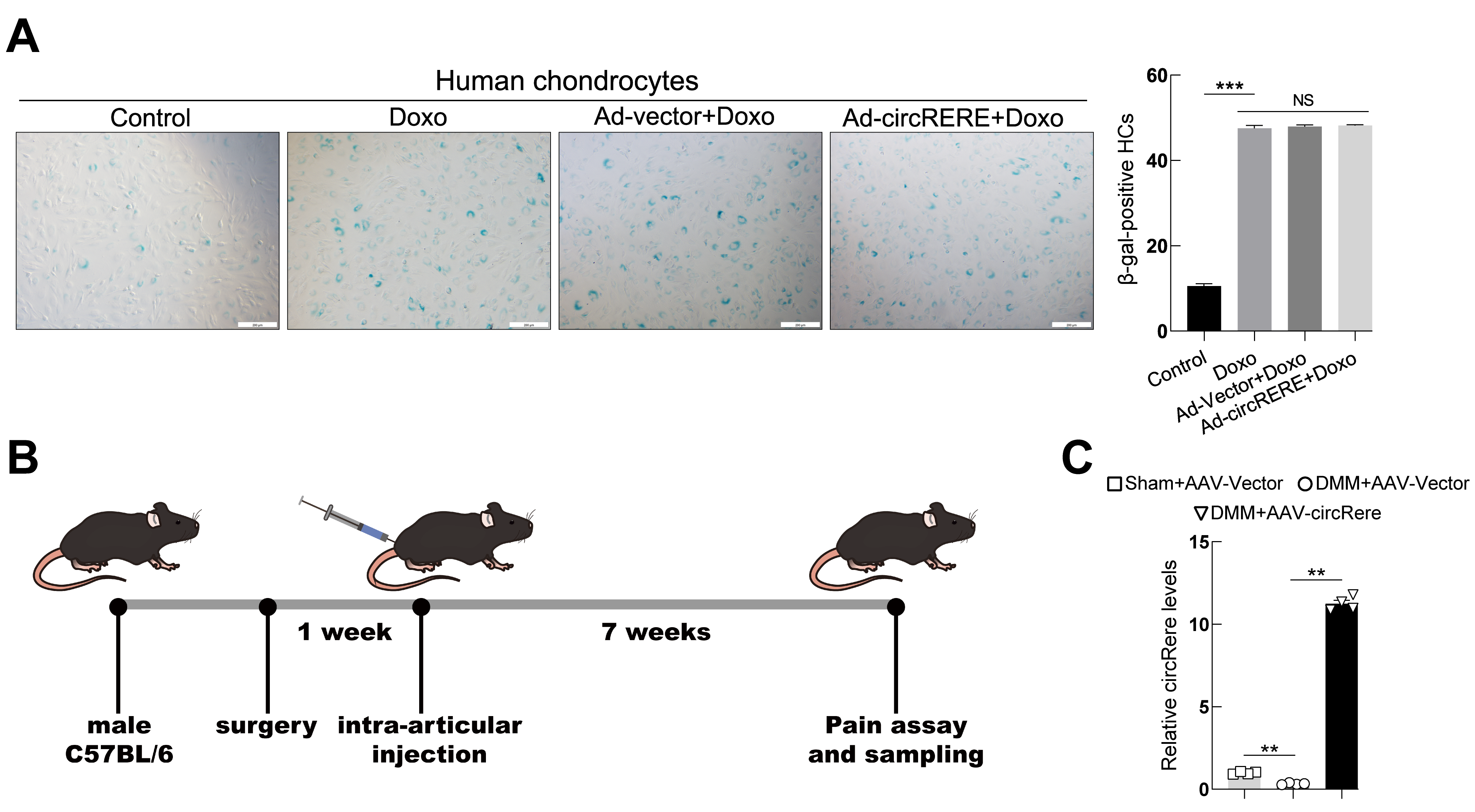
**

**FIGURE S2** (A) β-galactosidase staining and quantitative analysis for the β-gal-positive HCs infected with Ad-circRERE or Ad-vector and treated with doxorubicin (Doxo, 100 nM, 5 days). n=3. Scale bar, 200μm. NS, no significance. ***p<0.001 by One-way ANOVA with Tukey’s post hoc test. (B) Overview of DMM surgery, IA-injection with AAV-vector or AAV-circRere, pain assays and sampling. (C) Relative circRere expression in mouse cartilage from Sham+AAV-vector, DMM+AAV-vector and DMM+AAV-circRere groups. n=4. **p<0.01 by Brown-Forsythe and Welch ANOVA test followed by Dunnett’s T3 multiple comparison test. Data are presented as mean ± SEM.

**Figure S3**

**
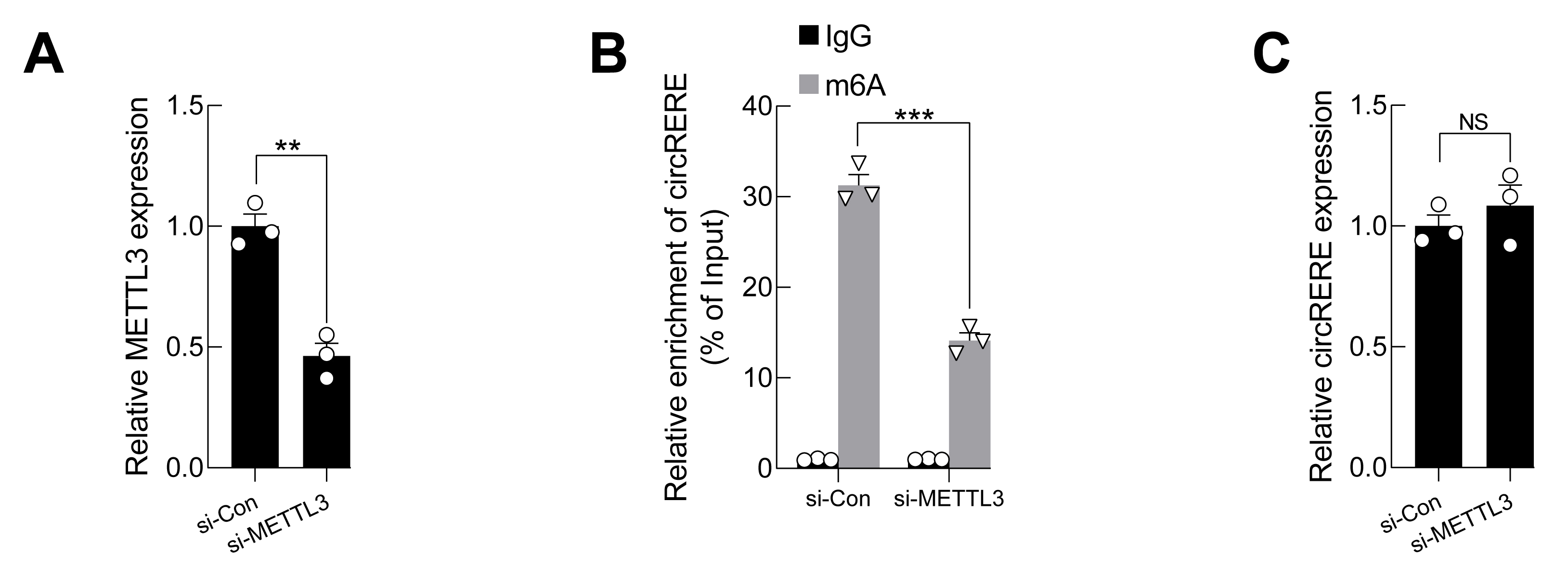
**

**FIGURE S3** (A) Efficiency of *METTL3* downregulation in human OA chondrocytes (isolated from damaged cartilage of OA patients) by si-METTL3 (n=3). **p<0.01 by two-tailed unpaired t test. (B) The percentage of m6A-modified circRERE upon METTL3 downregulation in human OA chondrocytes (isolated from damaged cartilage of OA patients) (n=3). ***p<0.05 by two-way ANOVA with Tukey’s post hoc test. (C) Relative expression of circRERE upon METTL3 downregulation in human OA chondrocytes (isolated from damaged cartilage of OA patients) (n=3). NS by two-tailed unpaired t test. Data are presented as mean ± SEM.

**Figure S4**

**
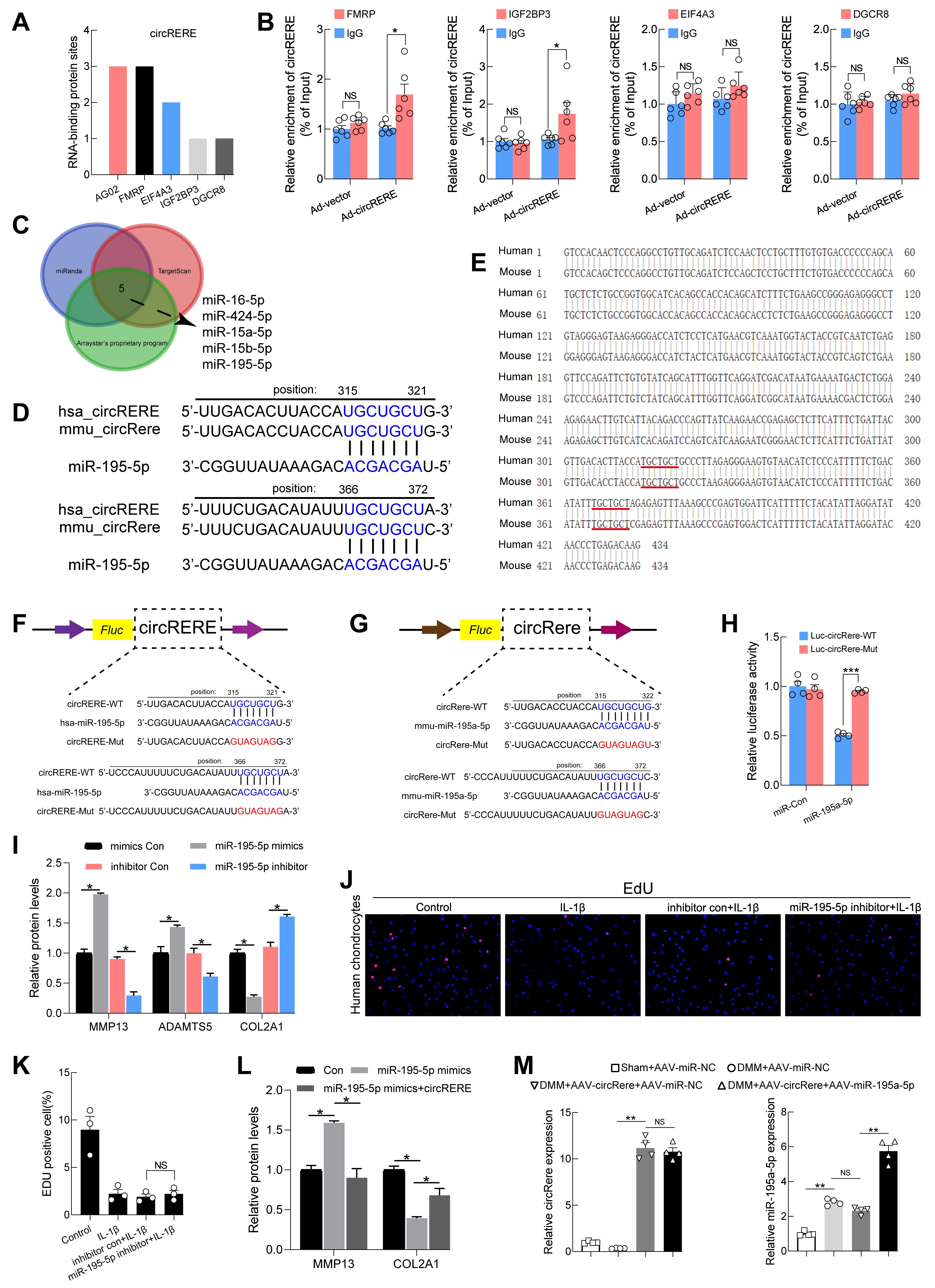
**

**FIGURE S4 (**A) Number of RBP sites between RBPs and circRERE. (B) FMRP, IGF2BP3, EIF4A3, and DGCR8 RIP assays and subsequent qRT-PCR analysis were performed to detect CO-RIPed-circRERE expression in HCs infected with Ad-vector or Ad-circRERE (n=6). *p<0.05, *p<0.01 by two-tailed unpaired t test or Welch’s t test. (C) Schematic to show the overlapping of the target miRNAs of circRERE, as predicted by miRanda, TargetScan and Arraystar’s proprietary program. (D) The potentially conserved binding sites between two circRNAs (hsa_circRERE, mmu_circRere) and miR-195-5p, predicted by miRanda and TargetScan. The potential complementary residues are shown in blue. (E) Pairwise sequence alignment of hsa_circRERE and mmu_circRere, and the sequences highlighted in red are targeted by miR-195-5p. (F) Schematic of luciferase reporter vectors containing wild-type (WT) or mutant (Mut) putative miR-195-5p binding sites of circRERE. (G) Schematic of luciferase reporter vectors containing wild-type (WT) or mutant (Mut) putative miR-195a-5p binding sites of mmu_circRere. (H) Relative luciferase activity of WT or MUT circRere luciferase reporter vector co-transfected with miR-195-5p mimics or negative control (n=4). ***p<0.001by two-tailed unpaired t test. (I) Quantification of Figure 4H (n=3). *p<0.05 by two-way ANOVA with Tukey’s post hoc test. (J, K) HCs were transfected with miR-195-5p inhibitor and stimulated with IL-1β (36h). Then proliferation was determined by EdU assays (n=3). One-way ANOVA with Tukey’s post hoc test. (L) Quantification of Figure 4L (n=3). *p<0.05 by two-way ANOVA with Tukey’s post hoc test. (M) Relative expressions of circRere and miR-195a-5p in knee cartilage from Sham+AAV-miR-NC, DMM+AAV-miR-NC, DMM+AAV-circRere+AAV-miR-NC and DMM+AAV-circRere+AAV-miR-195a-5p groups (n=4). **p<0.01 by Brown-Forsythe and Welch ANOVA test followed by Dunnett’s T3 multiple comparison test. NS, no significance. Data are presented as mean ± SEM.

**Figure S5**

**
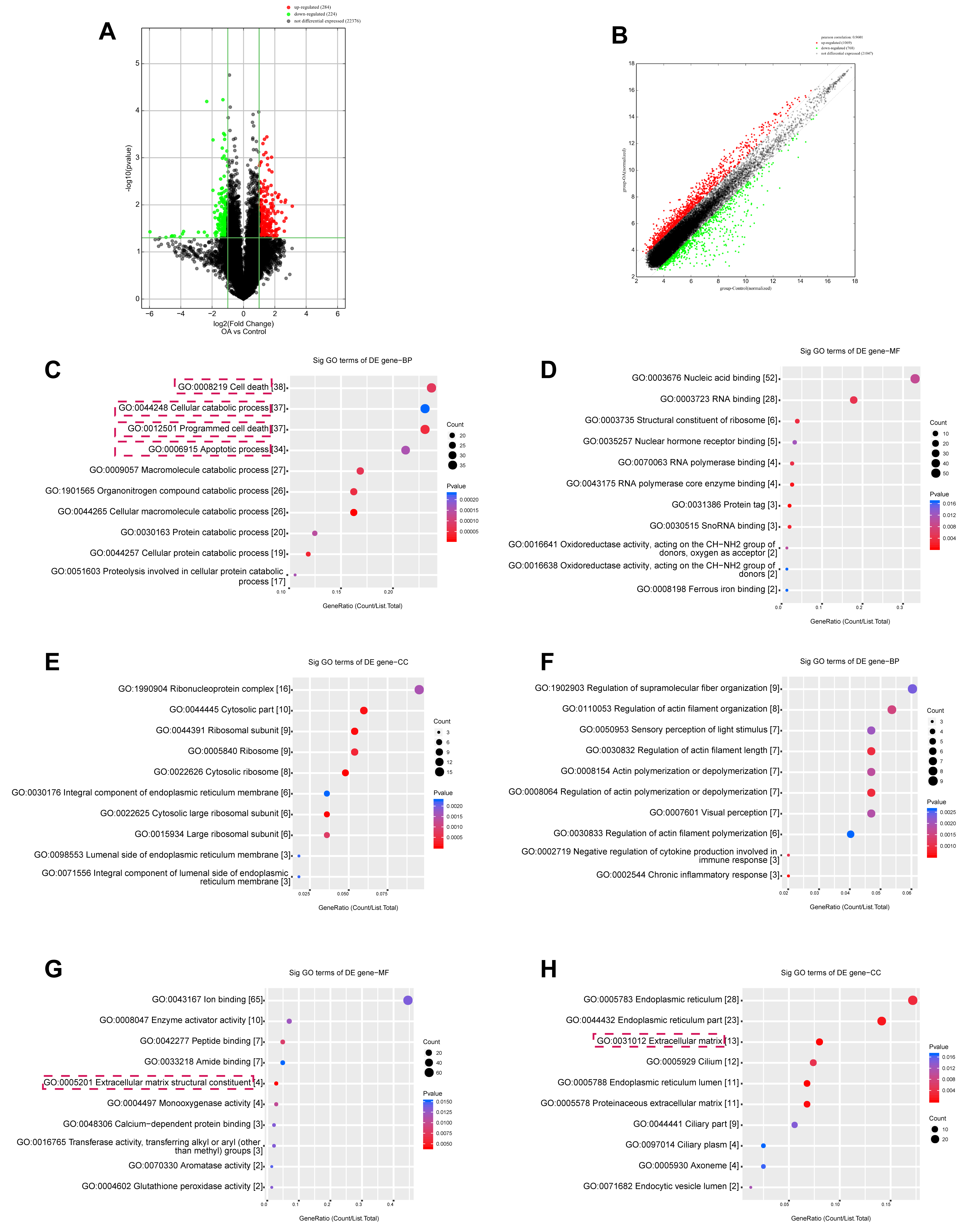
**

**
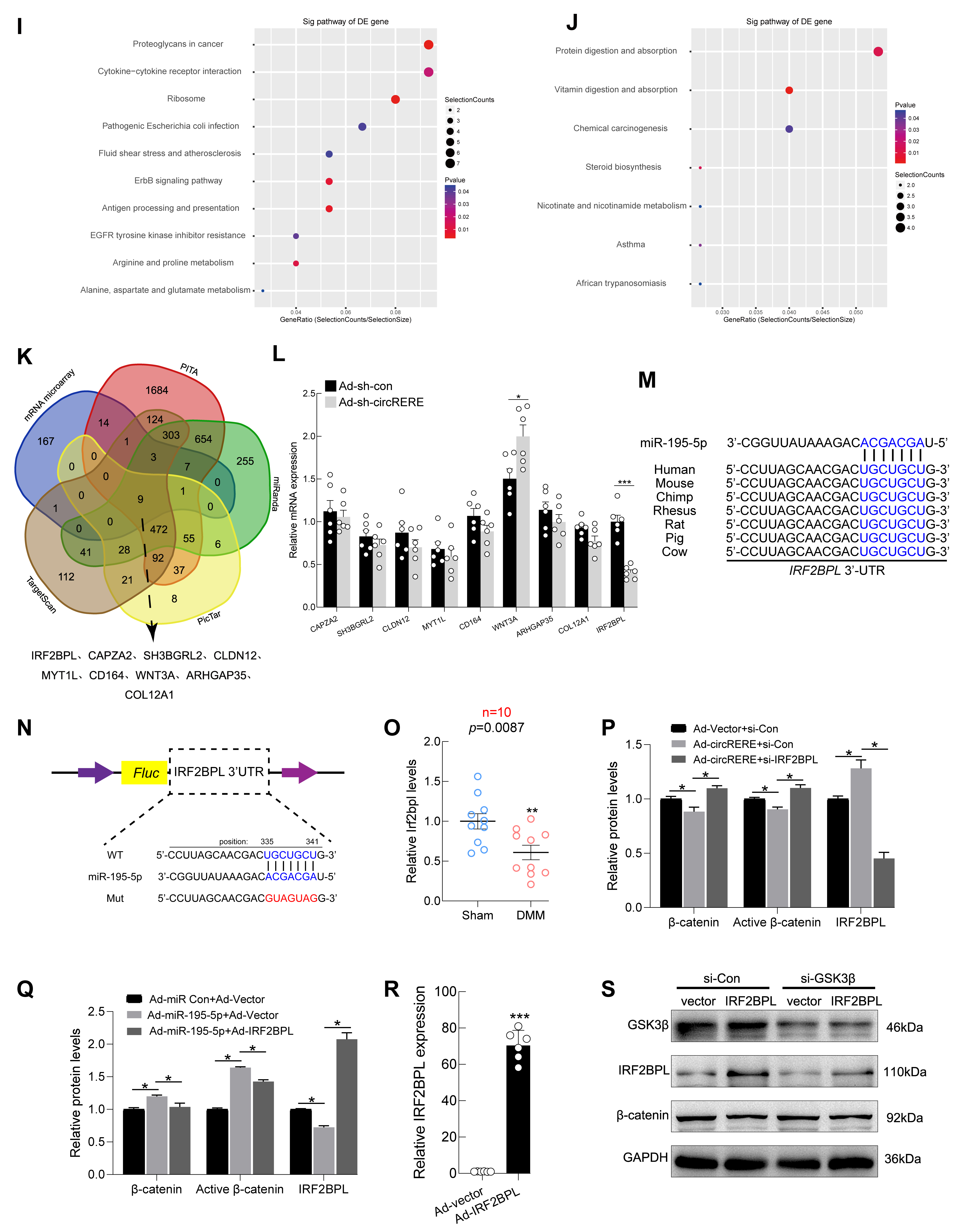
**

**FIGURE S5** (A) Volcano plot illustrating the statistical significance of differentially expressed mRNAs between human OA and control cartilage. (B) Scatter plot of differentially expressed mRNAs. (C-E) Gene Ontology (GO) analysis of upregulated genes in OA cartilage. (F-H) GO analysis of downregulated genes in OA cartilage. (I) Kyoto Encyclopedia of Genes and Genomes (KEGG) analysis of upregulated genes in OA cartilage. (J) KEGG analysis of downregulated genes in OA cartilage. (K) Schematic to show the overlapping of the target mRNAs of miR-195-5p, as predicted by PITA, miRanda, Pictar, TargetScan and downregulated mRNAs in OA cartilage identified by mRNA microarray. (L) QRT-PCR for relative expression of predicted target mRNAs of miRNA-195-5p in HCs infected with Ad-sh-con or Ad-sh-circRERE. n=6. ***p<0.001 by two-tailed unpaired t test. (M) Putative miR-195-5p binding sites in IRF2BPL. The potential complementary residues are shown in blue. (N) Schematic of luciferase reporter vectors containing wild-type (WT) or mutant (Mut) putative miR-195-5p binding sites of IRF2BPL 3’ UTR. (O) Relative expression of Irf2bpl in mouse cartilage from DMM and sham groups (n=10). **p<0.01 by two-tailed unpaired t test. (P, Q) Quantification of Figure 5Q (n=3). *p<0.05 by two-way ANOVA with Tukey’s post hoc test. (R) QRT-PCR for IRF2BPL expression in HCs infected with Ad-vector or Ad-IRF2BPL. n=6. ***p<0.01 by two-tailed unpaired t test. (S) WB analysis indicated that knockdown of GSK-3β did not abrogate the suppressive effect of IRF2BPL on β-catenin protein level in HCs. Data are presented as mean ± SEM.

**Figure S6**

**
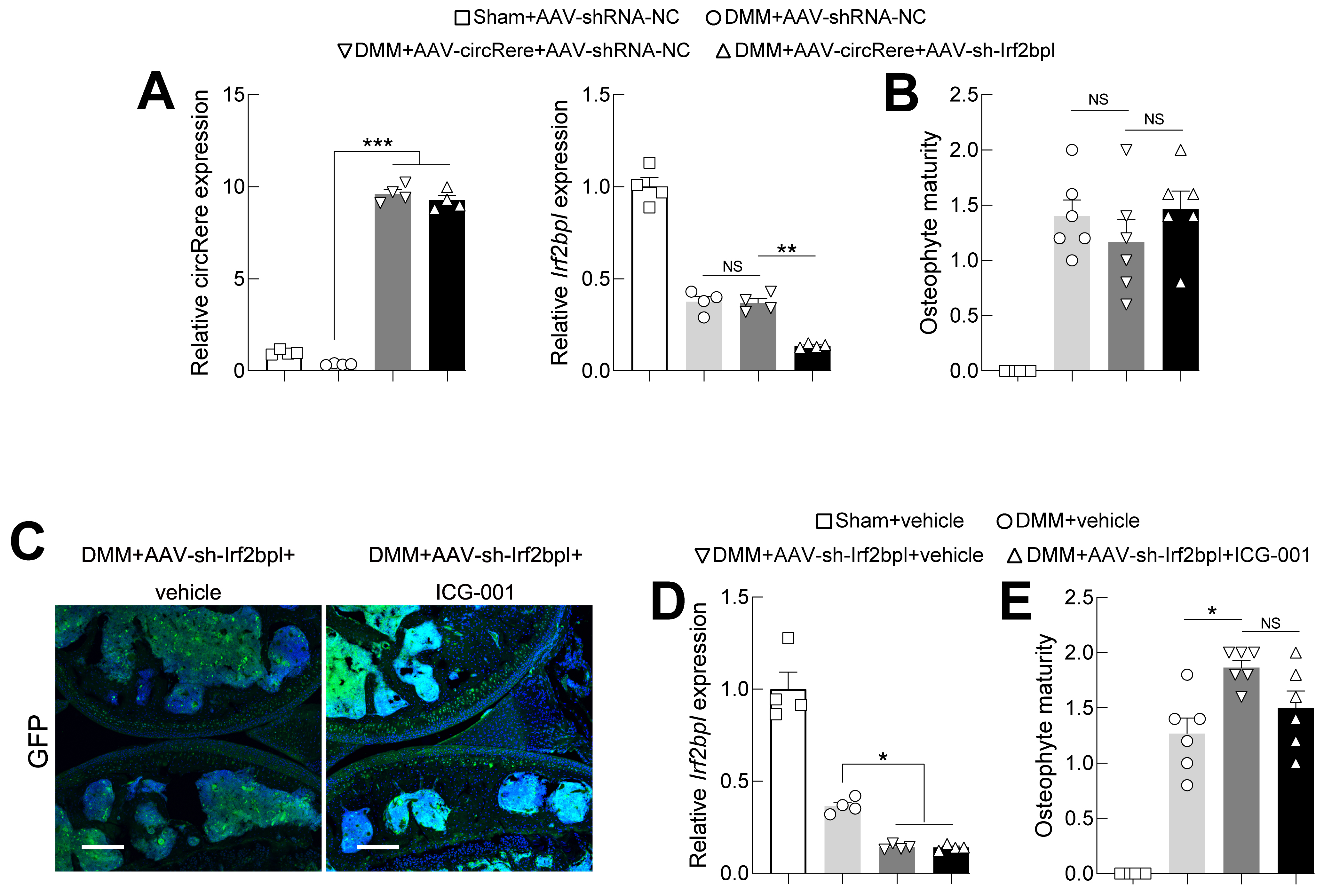
**

**FIGURE S6** (A) Relative expressions of circRere and *Irf2bpl* in knee cartilage from Sham+AAV-shRNA-NC, DMM+AAV-shRNA-NC, DMM+AAV-circRere+AAV-shRNA-NC and DMM+AAV-circRere+AAV-sh-Irf2bpl groups (n=4). **p<0.01, ***p<0.001 by one-way ANOVA with Tukey’s post hoc test. (B) Scoring of osteophyte maturity in four groups. one-way ANOVA with Tukey’s post hoc test. (C) To investigate the infected efficiency of AAVs, representative knee cartilage fluorescence (GFP) images in knee sections from DMM+AAV-sh-Irf2bpl+vehicle and DMM+AAV-sh-Irf2bpl+ICG-001 groups were obtained by a confocal microscope. Scale bar, 200μm. (D) Relative expression of *Irf2bpl* in knee cartilage from Sham+vehicle, DMM+vehicle, DMM+AAV-sh-Irf2bpl+vehicle and DMM+AAV-sh-Irf2bpl+ICG-001 groups (n=4). *p<0.05 by one-way ANOVA with Tukey’s post hoc test. (E) Scoring of osteophyte maturity in four groups (n=6). *p<0.05 by one-way ANOVA with Tukey’s post hoc test. NS, no significance. Data are presented as mean ± SEM.

**Supplementary Table S1. The primers and sequences used in this study**

| Primers for RT-PCR | | |  |
| --- | --- | --- | --- |
| circFMN2 (human) #1 | F | TGTGAAGTTGAAGCAGGGAAAG | |
|  | R | ACTGTTCAGGTTTGTCCAGAGA | |
| circFMN2 (human) #2 | F | ACCAGGTCTCCTCAAAAGAGC | |
|  | R | TTCGAGTTCGTCTGACTGTGC | |
| circFMN2 (human) #3 | F | CCTGTGAAGTTGAAGCAGGGA | |
|  | R | TCGAGTTCGTCTGACTGTGC | |
| circTLK1 (human) #1 | F | CAAGTGGGAGCACTGGAAGT | |
|  | R | GCCTTCTTGGATCCAGACTATGA | |
| circTLK1 (human) #2 | F | GGGAGCACTGGAAGTACGG | |
|  | R | TCCAGACTATGAAGCTCATCCAT | |
| circTLK1 (human) #3 | F | TTACTGGAGTTGCAAGTGGG | |
|  | R | TAGCTTCCAATAACTCTTGCCT | |
| circDENND1A (human) #1 | F | CAGAGATTCGGGTTCTGCCG | |
|  | R | GTCCACATAGAAGGGGAAACAA | |
| circDENND1A (human) #2 | F | TGCCGCTTATCTTCAGGAGC | |
|  | R | TGTCCACATAGAAGGGGAAACA | |
| circDENND1A (human) #3 | F | ATCTTCAGGAGCGAAGAGCTG | |
|  | R | GAAGGGGAAACAAAACTTGGTC | |
| circSMARCB1 (human) #1 | F | CGGATACACGACTCTAGCCA | |
|  | R | GAGAACCTCGGAACATACGG | |
| circSMARCB1 (human) #2 | F | ATACACGACTCTAGCCACCAG | |
|  | R | CCAGAGTGAGGGGTATCTCTTG | |
| circSMARCB1 (human) #3 | F | ACGATGAGAAGTACAAGGCTG | |
|  | R | AGAACCTCGGAACATACGGA | |
| circRNF114 (human) #1 | F | CGGCACCGGTTTTCTTATGAC | |
|  | R | TGCATCCTTAATGGTGGCCTT | |
| circRNF114 (human) #2 | F | CTGGGGAGACCCCAACTACC | |
|  | R | GACCGGATCTTGGACAGGAAG | |
| circRNF114 (human) #3 | F | GGTTTGTCCGATATGTGCCTC | |
|  | R | CCTTAATGGTGGCCTTCACAC | |
| circZFHX4 (human) #1 | F | ACGCTTGCATACCACCAATCA | |
|  | R | TCACTTTTGCGCTCATCCGT | |
| circZFHX4 (human) #2 | F | ACGCTTGCATACCACCAATC | |
|  | R | CACTTTTGCGCTCATCCGTT | |
| circZFHX4 (human) #3 | F | ATTGGCAACCCTGTTCACCT | |
|  | R | CAAGGCTTCACTTTTGCGCT | |
| circLDLRAD4 (human) #1 | F | CGATGGACTTGGACAGGCTA | |
|  | R | GCCCAACAGGAGGTTTGATG | |
| circLDLRAD4 (human) #2 | F | AGCAGAGCGATGGACTTGGA | |
|  | R | TGATGGGAGTTCTCGGAAATGG | |
| circLDLRAD4 (human) #3 | F | AAGCTGGTTTTCAGGCCACA | |
|  | R | TCTCGGAAATGGCCCAACAG | |
| circTENM3 (human) #1 | F | AGCAACTCGAAGAGGACTGG | |
|  | R | ACATCAGTCCTGCTGGTGAC | |
| circTENM3 (human) #2 | F | GAGGACTGGCATTTTGTGCG | |
|  | R | GCCTGCGTTCTTTCACATCC | |
| circTENM3 (human) #3 | F | CAACTCGAAGAGGACTGGCAT | |
|  | R | AGGCTCAGGGTTCAAGTCTC | |
| circRMDN2 (human) #1 | F | ACCAGAATGCTTTGAAGTTCTGT | |
|  | R | CTCTCCGACTTGCCAGACTC | |
| circRMDN2 (human) #2 | F | TGATCTTGAGGAAAACCAGAATGC | |
|  | R | CTCCGACTTGCCAGACTCAC | |
| circRMDN2 (human) #3 | F | TGGCTTTATTGCTTCCTACTGTT | |
|  | R | TCAAAACTCTCCGACTTGCCA | |
| circFRAS1 (human) #1 | F | GTGGGCTGGAGTGCTCATC | |
|  | R | TTTCCAGGGACTCGGACTAC | |
| circFRAS1 (human) #2 | F | GGGGACGGCTTCTACCAAGA | |
|  | R | ACATTTTCCAGGGACTCGGA | |
| circFRAS1 (human) #3 | F | TGGGGACGGCTTCTACCAAG | |
|  | R | CAACATTTTCCAGGGACTCGG | |
| circRERE (human) #1 | F | CCAGTTATCAAGAACCGAGAGC | |
|  | R | GTCACACAAAGCAGGAGTTGG | |
| circRERE (human) #2 | F | GTTGACACTTACCATGCTGCT | |
|  | R | TCTCCCGGCTTCAGAAAGATG | |
| circRERE (human) #3 | F | CTGCTAGAGAGTTTAAAGCCCG | |
|  | R | TCTCCCGGCTTCAGAAAGATG | |
| circTBCK (human) #1 | F | GGCAGAAGTTCGGCACCTTA | |
|  | R | AGCTCTGGATCATGAAATGCAATC | |
| circTBCK (human) #2 | F | ACAGCATGCTCAACCTCCAA | |
|  | R | AGCTCTGGATCATGAAATGCAA | |
| circTBCK (human) #3 | F | CCAAAGCCATCTTCTGACAGC | |
|  | R | TGGATCATGAAATGCAATCATCTG | |
| circARHGAP5 (human) #1 | F | ATCCTGCAATCACTTCTGACCA | |
|  | R | TGGTATAGGATGGGGGACGAG | |
| circARHGAP5 (human) #2 | F | TCCTGCAATCACTTCTGACCA | |
|  | R | GGGGACGAGGCTCTTTGTTT | |
| circARHGAP5 (human) #3 | F | TGTTGAAACTTGGAAAGGTGGT | |
|  | R | GATGGTATAGGATGGGGGACG | |
| RERE (human) | F | AGAGAACTTGTCATTACAGACCCA | |
|  | R | GCAGCAGCATGGTAAGTGTC | |
| Divergent-GAPDH (human) | F | GAAGGTGAAGGTCGAGTC | |
|  | R | GAAGATGGTGATGGGATTTC | |
| GAPDH (human) | F | TGCACCACCAACTGCTTAGC | |
|  | R | GGCATGGACTGTGGTCATGAG | |
| β-actin (human) | F | AGAGCTACGAGCTGCCTGAC | |
|  | R | AGCACTGTGTTGGCGTACAG | |
| circRere (mouse) #1 | F | GACATATTTGCTGCTCGAGAGTT | |
|  | R | GAGCTGGAGATCTGCAACAGG | |
| circRere (mouse) #2 | F | CGAGAGTTTAAAGCCCGAGTG | |
|  | R | GGGACTTCAGACTGACGGTA | |
| circRere (mouse) #3 | F | CCTACCATGCTGCTGCCCTA | |
|  | R | TGGGACTTCAGACTGACGGTA | |
| Rere (mouse) | F | AGGGACCATCTACTCATGAACG | |
|  | R | GCCGATCCTGAACCAAATGC | |
| GAPDH (mouse) | F | AGGTCGGTGTGAACGGATTTG | |
|  | R | TGTAGACCATGTAGTTGAGGTCA | |
| β-actin (mouse) | F | TGAGCTGCGTTTTACACCCT | |
|  | R | TTTGGGGGATGTTTGCTCCA | |
| β-catenin (human) | F | TCCTGAGGAAGAGGATGTGGAT | |
|  | R | CCTCTGAGCTCGAGTCATTGC | |
| RARA (human) | F | GGGGGAATCCTGAATCGAGC | |
|  | R | GATGTCTCAAGAGCCGGTCC | |
| hsa-miR-424-5p | RT | Ribobio | |
|  | F | Ribobio | |
|  | R | Ribobio | |
| hsa-miR-195-5p | RT | Ribobio | |
|  | F | Ribobio | |
|  | R | Ribobio | |
| hsa-miR-15a-5p | RT | Ribobio | |
|  | F | Ribobio | |
|  | R | Ribobio | |
| hsa-miR-15b-5p | RT | Ribobio | |
|  | F | Ribobio | |
|  | R | Ribobio | |
| hsa-miR-16-5p | RT | Ribobio | |
|  | F | Ribobio | |
|  | R | Ribobio | |
| U6 | RT | Ribobio | |
|  | F | Ribobio | |
|  | R | Ribobio | |
| IRF2BPL (human) | F | TCCCGGAGACAATCTTGCTA | |
|  | R | CATACGGGTTCCGAGAAGTC | |
| Irf2bpl (mouse) | F | ACTCAGATCGTAGGGGGACC | |
|  | R | CTGGTCCAGAGAAGAGCACC | |
| CAPZA2 (human) | F | GACAGTTGCCAGTTACACGC | |
|  | R | AATGGCAACAGCCCATCTGA | |
| SH3BGRL2 (human) | F | AGAGGATGGTCATCCGCGTG | |
|  | R | GGTTGCCCTGAGTGGGTTTC | |
| CLDN12 (human) | F | CGAGTAAAATGCCCTGCGTG | |
|  | R | AACTTCCCTGTGCCTCGTTT | |
| MYT1L (human) | F | CCCTGCAGTCTTGTTGAAGC | |
|  | R | GACACATGCATGCCACCAAG | |
| CD164 (human) | F | CTTCTGTTCCGCTAAACCCAC | |
|  | R | CACAGGTTGTGAGGTTGGAGT | |
| ARHGAP35 (human) | F | GCCCACCTTGATGAGACCTG | |
|  | R | GGTGATGGGCCGATTGTAGA | |
| COL12A1 (human) | F | CACAGGTTCAAGAGGTCCCC | |
|  | R | ATGTGTTAGCCGGAACCTGG | |
| Probes for FISH | | |  |
| FAM-hsa-miR-195-5p | Ribobio | |  |
| FAM-mmu-miR-195a-5p | Ribobio | |  |
| Cy3-circRERE (human) | Ribobio | |  |
| Cy3-circRere (Mouse) | Ribobio | |  |
| siRNA /shRNA | | |  |
| siRNA NC | GGCUCUAGAAAAGCCUAUGC | |  |
| human circRERE siRNA#1 | AACCCTGAGACAAGGTCCACA | |  |
| human circRERE siRNA#2 | CCCTGAGACAAGGTCCACA | |  |
| human circRERE siRNA#3 | TGAGACAAGGTCCACAACT | |  |
| Mouse circRere shRNA | AACCCTGAGACAAGGTCCACA | |  |
| human IRF2BPL siRNA | GCATCGAATTCGTGATCGA | |  |
| human METTL3 siRNA | GCAAGUAUGUUCACUAUGA | |  |
| human YTHDF2 siRNA | AAGGACGUUCCCAAUAGCCAA | |  |
| human HRSP12 siRNA | UGUAAUAGGGAGAGUUGAA | |  |
| human POP1 siRNA | GAAUUUAACCGUAGACAAA | |  |
| Mouse Ythdf2 siRNA | AAGGACGUUCCCAAUAGCCAA | |  |
| Mouse Irf2bpl shRNA | GGACTATGAGCTGAAGCTGTT | |  |
| Mimics/Inhibitor | | |  |
| hsa-miR-195-5p mimics-sense | UAGCAGCACAGAAAUAUUGGC | |  |
| hsa-miR-195-5p mimics-anti-sense | GCCAAUAUUUCUGUGCUGCUA | |  |
| mimics Con-sense | UUUGUACUACACAAAAGUACUG | |  |
| mimics Con-anti-sense | CAGUACUUUUGUGUAGUACAAA | |  |
| hsa-miR-195-5p inhibitor | GCCAAUAUUUCUGUGCUGCUA | |  |
| inhibitor Con | CAGUACUUUUGUGUAGUACAAA | |  |
